# Machine Learning and Feature Selection in Pediatric Appendicitis

**DOI:** 10.3390/tomography11080090

**Published:** 2025-08-13

**Authors:** John Kendall, Gabriel Gaspar, Derek Berger, Jacob Levman

**Affiliations:** 1 Department of Computer Science, St. Francis Xavier University, Antigonish, NS B2G 2W5, Canadax2020eqg@stfx.ca (G.G.);; 2 Nova Scotia Health Authority, Halifax, NS B3H 1V8, Canada

**Keywords:** appendicitis, pediatrics, predictive medicine, machine learning, classification

## Abstract

Background/Objectives: Accurate prediction of pediatric appendicitis diagnosis, management, and severity is critical for clinical decision-making. We aimed to evaluate the predictive performance of a wide range of machine learning models, combined with various feature selection techniques, on a pediatric appendicitis dataset. A particular focus was placed on the role of ultrasound (US) image-descriptive features in model performance and explainability. Methods: We conducted a retrospective cohort study on a dataset of 781 pediatric patients aged 0–18 presenting to Children’s Hospital St. Hedwig in Regensburg, Germany, between January 2016 and February 2023. We developed and validated predictive models; machine learning algorithms included the random forest, logistic regression, stochastic gradient descent, and the light gradient boosting machine (LGBM). These were paired exhaustively with feature selection methods spanning filter-based (association and prediction), embedded (LGBM and linear), and a novel redundancy-aware step-up wrapper approach. We employed a machine learning benchmarking study design where AI models were trained to predict diagnosis, management, and severity outcomes, both with and without US image-descriptive features, and evaluated on held-out testing samples. Model performance was assessed using overall accuracy and area under the receiver operating characteristic curve (AUROC). A deep learner optimized for tabular data, GANDALF, was also evaluated in these applications. Results: US features significantly improved diagnostic accuracy, supporting their use in reducing model bias. However, they were not essential for maximizing accuracy in predicting management or severity. In summary, our best-performing models were, for diagnosis, the random forest with embedded LGBM feature selection (98.1% accuracy, AUROC: 0.993), for management, the random forest without feature selection (93.9% accuracy, AUROC: 0.980), and for severity, the LGBM with filter-based association feature selection (90.1% accuracy, AUROC: 0.931). Conclusions: Our results demonstrate that high-performing, interpretable machine learning models can predict key clinical outcomes in pediatric appendicitis. US image features improve diagnostic accuracy but are not critical for predicting management or severity.

## 1. Introduction

Pediatric appendicitis is characterized by inflammation of the appendix found in patients aged eighteen years and younger. When inflamed, the appendix causes pain and can lead to serious complications for the patient, including peritonitis and infection [1]. Symptoms can include nausea, loss of appetite, constipation, bloating, and abdominal pain [1]. Symptoms are not always easily identified or caught in time in younger patients, as they may not communicate as well and often experience fewer symptoms [2]. Appendicitis is typically caused by a blockage in the lumen, leading to an infection that then causes the appendix to expand and potentially burst [1]. While appendicitis can occur in both males and females, males have been found to be at a slightly higher risk, and most cases occur between the ages of ten and thirty [1]. A highly effective way to diagnose appendicitis is to evaluate the current state of the appendix using medical imaging. This is performed through computed tomography (CT), ultrasound (US), or magnetic resonance imaging (MRI), with CT being the most accurate of the three [3]. A shortcoming of these imaging techniques is that they are expensive and potentially time-consuming. MRI may not always be readily accessible due to high costs, limited availability, and the need for specialized interpretation, all of which can delay diagnosis and treatment. Additionally, CT relies upon ionizing radiation, which for most adults is safe, but may be risky for younger patients due to the radiation’s potential negative effects on their growing bodies [3].

Supervised machine learning is a common technology applied to predictive applications, such as diagnosing a given medical condition. The algorithms are provided with ground-truth training data, which are represented by sets of samples/instances, each containing a set of feature measurements that can inform predictions, and a target variable to be predicted. During training, algorithms establish complex correlational relationships between predictor variables and the target variable, supporting the creation of technologies that can be relied upon to make predictions on samples that were not trained upon. As such, as long as correlations exist between predictor variables and the target variable, AI has the potential to create highly accurate predictive models.

Using artificial intelligence technologies to diagnose appendicitis has been the subject of previous analyses. One study from Saudi Arabia used K-nearest neighbours (KNN), decision trees (DT), bagging, and stacking to identify acute appendicitis and found their stacking model to be the most successful with training accuracy, testing accuracy, testing precision, and testing F1 scores of 97.51%, 92.63%, 95.29%, and 92.04%, respectively [4]. From their study, they found their most important features to be neutrophils, white blood cell count, length of stay, and symptom days for their stacking model [4]. Another study [5] was conducted using results from previous studies [6,7,8,9,10,11,12,13,14,15,16,17,18,19,20,21,22,23,24,25,26,27,28,29,30,31,32,33,34] to determine whether using AI models is an effective way for diagnosing acute appendicitis in adults. This review analyzed twenty-nine studies on diagnosis and prognosis of acute appendicitis, and found that the model most commonly used was the artificial neural network (ANN) [5]. These ANNs produced accuracy scores typically above 80% with some reporting the area under the receiver operating characteristic curve (AUC) nearing 0.99 [5]. However, it should be noted that this analysis was based on an adult population, and so the findings therein may not hold in a pediatric population.

Several recent studies have applied machine learning approaches to pediatric appendicitis using subsets of the dataset analyzed in the present work. A foundational study [35] on a subset of the dataset addressed in this research [36] was previously conducted and included 430 patients. The machine learning models used were logistic regression, random forest, and generalized boosted regression model, all in the R programming language. Their results are summarized: “A random forest classifier achieved areas under the precision-recall curve of 0.94, 0.92, and 0.70, respectively, for the diagnosis, management, and severity of appendicitis”, based on held-out test samples as part of 10-fold validation [35]. A subsequent analysis, as part of a larger team, was performed using a larger subset (579 patients) of the dataset addressed in this study to diagnose pediatric appendicitis using deep learning methods with concept bottleneck models (CBMs) with a primary focus on the ultrasound images [37]. While the dataset contains images and corresponding descriptions of the images, some patients included do not contain a complete set of all of these features. The images are taken from multiple views of the same target area to help ensure imaging has captured key features of the appendix being analyzed. To handle this, the study used a semi-supervised extension in addition to the CBM [37]. They first used a shared encoder neural network to map the images to features, which are then aggregated across imaging views to produce representations and concepts understandable by humans, contributing to the prediction of the target class [37]. Results of 0.80 AUROC were reported for predicting the diagnosis of appendicitis. Two additional studies have been conducted on the updated dataset used in this analysis, focused exclusively on diagnosis [38,39]. This includes an approach achieving 94.5% accuracy with the random forest [38], and an approach based on the Hybrid Bat algorithm achieving 94% accuracy [39]. An additional analysis focused on diagnosis and severity [40] but did not report accuracy statistics.

### Hypotheses and Contributions

Our objective in this study is to address the following hypotheses. We hypothesize that:

▪ The use of open-source machine learning software applied to the Regensburg Pediatric Appendicitis Dataset may produce useful technology for predicting aspects of pediatric appendicitis patient care.
▪ By creating technologies that can predict diagnosis, severity, and management of pediatric appendicitis, both by using and withholding US image-derived features, we can assess the apparent value of US imaging in the context of AI predictive technology.
▪ Our models will be able to more accurately predict their respective target variables (diagnosis, management, and severity), as compared to previous works on this topic, by thoroughly examining a large set of combinations of machine learning and feature selection algorithms.
▪ Feature selection subsets will be informative to clinicians and researchers as to factors that are predictive of diagnosis, management, and severity of pediatric appendicitis, respectively.

Contributions provided by this study include the consideration of a large selection of feature selection (FS) algorithms, including a novel redundancy-aware FS algorithm developed in our lab, consideration of novel subsets of features identified by FS, consideration of a variety of high-performing machine learning algorithms, including the computationally efficient light gradient boosting machine and a deep learner optimized for tabular data, known as Gandalf, evaluation of our study findings on an updated pediatric appendicitis dataset with more patients/samples than those included in the early work on this topic, confirmation of the value of ultrasound imaging features as assisting in mitigating bias in prediction for diagnosis of appendicitis, and finally, demonstrating strong predictive performance from the models developed across three AI applications in pediatric appendicitis.

We introduced an overview of pediatric appendicitis, related AI technological development, and closely related work on the same dataset in Section 1, as well as provided a Hypotheses and Contributions subsection. The rest of the paper will proceed as follows: we will provide a study design overview in Section 2.1, an outline of the study participants in Section 2.2, a detailed dataset description of the variables/measurements in Section 2.3, an outline of the preprocessing performed on the dataset in Section 2.4, the machine learning methods used are presented in Section 2.5, and the statistics relied upon for machine learning evaluation are presented in Section 2.6. The results for predicting diagnosis are provided in Section 3.1, the results for predicting management are provided in Section 3.2, the results for predicting severity are provided in Section 3.3, and the Gandalf deep learner results are provided in Section 3.4. A discussion of interactions between machine learning and feature selection technologies employed is provided in Section 4.1, a discussion of Gandalf results is provided in Section 4.2, a discussion of the value of Ultrasound features is provided in Section 4.3, a literature comparison is provided in Section 4.4, future work is presented in Section 4.5, followed by our conclusions in Section 5.

## 2. Materials and Methods

### 2.1. Study Design Overview

We conducted a retrospective cohort study on a dataset of 781 pediatric patients aged 0–18 presenting to Children’s Hospital St. Hedwig in Regensburg, Germany, between January 2016 and February 2023. This study employed a comparative AI benchmarking approach using publicly available benchmarking software applied to an open-access pediatric appendicitis dataset. The analysis covered three clinical tasks: diagnosis (the AI is tasked with performing a diagnosis of appendicitis or not), management (the AI is tasked with predicting the treatment option for the patient), and severity (the AI is tasked with predicting the state of the patient’s appendicitis). The potential value from the inclusion of ultrasound image features was considered for all applications. This study was performed retrospectively on a public domain dataset; as such, no ethics committee approval was required for this analysis.

### 2.2. Participants

The dataset examined was initially assembled by Marcinkevičs et al., and their analysis was previously published [35]. The dataset was revisited [37] with an extended observation timeline, more patients, and additionally collected ultrasound images for many of the patients. The dataset previously studied [37] included records for 579 patients, whereas we examined an updated version of this dataset with 781 observations. The data was obtained from patients admitted to the tertiary Children’s Hospital St. Hedwig in Regensburg, Germany, with suspected appendicitis between 2016 and 2021. All aspects of the methods of this study were completed by the study authors except for the patient recruitment and data acquisition/curation previously completed [35,37].

### 2.3. Variables/Measurements

Patient data included demographic information, clinical examinations, laboratory tests, scoring results, and (potentially multiple per patient) ultrasound (US) images and expert-interpreted findings from the images. Descriptions of the feature measurements and target variables are detailed in Table 1, Table 2 and Table 3, and their numeric feature distributions in Table 4. The categorical feature statistics tables have also been provided in Appendix A (Table A1, Table A2 and Table A3). Detailed feature descriptions are also provided in Appendix A, broken down for different feature types, see Table A4, Table A5, Table A6, Table A7, Table A8 and Table A9. Note that there was a single patient/sample with a missing diagnosis field in this dataset; as such, it needed to be excluded from the diagnosis application, resulting in a count of 780 samples for the diagnosis application, whereas we were able to maintain the full sample count of 781 for the remaining two target variable applications. Predictive models were created to target the same three variables previously targeted [35] for binary classification:

- Diagnosis: Appendicitis (*n* = 463, 59.36%) or no appendicitis (*n* = 317, 40.64%).
- Management: Surgical (*n* = 298, 38.16%) or conservative (*n* = 483, 61.84%).
- Severity: Complicated (*n* = 119, 15.24%) or uncomplicated (*n* = 662, 84.76%).

### 2.4. Data Preprocessing

Df-analyze, the software relied upon for our machine learning and feature selection analysis, performs its own data cleaning [41], so null value handling was left to its imputation feature with median selection. A variety of preprocessing steps were applied prior to the use of df-analyze. The US number was dropped as it acted as a unique ID. All urine sample features were converted from categorical features to an ordinal scale from 0 to 3, so the relationship between values was encapsulated in the feature encodings. The management target variable was reduced to a binary class by combining primary surgical, secondary surgical, and simultaneous appendectomy in a single surgical class, as df-analyze requires substantial class representation for all target values for its validation to function. The data summary suggests secondary surgical management indicates surgery after their initial stay, when the patient data was recorded. As part of the previous analysis [35], patients were contacted at least 6 months after discharge and classified their management as (secondary) surgical if they had since had an appendectomy. As was previously investigated [35], we predict whether a patient required surgery, as it could potentially prevent a second visit to the hospital. Length of stay was also dropped from the dataset, as we have created technologies with potential real-world utility, in which we would want to be able to predict important target variables, such as diagnosis, severity, and management as early on in their hospital admission as possible, and we cannot establish the correct length of stay value for each patient until the end of their hospitalization.

The presumptive diagnosis feature may not always match the final diagnosis and may provide additional information reinforced by the managing doctors’ education and expertise, which could be particularly useful in smaller datasets. However, the feature may bias a machine learning model, or in real-world applications, may not be available for input. As such, this feature was excluded from our dataset.

Lymph Nodes Location, Abscess Location, and Gynecological findings were excluded from our dataset, as they were all described as free-form text, mostly in German. When divided into classes by unique values, Abscess Location and Gynecological findings’ largest class had fewer than 20 instances, which is too few for informing reliable predictions in df-analyze [41]. Lymph Nodes Location had some unique values with at least 20 instances, but many of its classes were combinations of others, and the feature is null for more than 80% of records; as such, it was also excluded. To facilitate reproducibility, custom pre-processing code for this dataset is provided in clean-tabular-dataset.py [42].

### 2.5. Machine Learning

The machine learning software used in this study is df-analyze [41]. The models considered in this study include the light gradient boosting machine (LGBM), random forest (RF), linear regression (LR), stochastic gradient descent (SGD), k-nearest neighbours (KNN), and a dummy model that predicts the class with the largest number of samples as a baseline. Df-analyze also supports assessment of a variety of feature selection (FS) technologies [41], each of which is exhaustively combined with all supported aforementioned machine learning methods. This includes two types of filter-based FS: association (assoc) and prediction (pred) [41], two types of embedded FS: linear (embed_linear) and LGBM (embed_lgbm) [41], and an emerging redundancy-aware step-up feature selection method (wrap) unique to df-analyze [43], as well as no (none) FS. The target features in this study were predicted from exhaustive combinations of supported machine learning and FS algorithms trained and tested individually as part of a fair comparison validation. For each target variable, models are constructed with each FS method. Optuna hyperparameter tuning is supported in df-analyze [41] and was used in this analysis for all machine learning techniques.

The code for running all configurations of our dataset with command line interfaces (CLIs) is provided in run-df-analyze.sh [42]. Each target variable was run with and without US image features. Thus, our analysis involves six runs of df-analyze as follows:

- Targeting Diagnosis with US Image Features Included;
- Targeting Diagnosis without US Image Features Included;
- Targeting Management with US Image Features Included;
- Targeting Management without US Image Features Included;
- Targeting Severity with US Image Features Included;
- Targeting Severity without US Image Features Included.

### 2.6. Statistical Analysis

Df-analyze conducts statistical analyses of each classification model paired with each FS method, using eight different metrics. These metrics are: overall accuracy (acc—the proportion of correct predictions out of all predictions), balanced accuracy (bal-acc—the expected accuracy if the dataset classes were balanced), F1-score (f1—the harmonic mean of recall and precision), negative predicted value (npv—the proportion of negative predictions that are correct), positive predicted value (ppv—the proportion of positive predictions that are correct), sensitivity (sens—the proportion of the group of interest predicted correctly), specificity (spec—the proportion of the group not-of-interest predicted correctly), and the area under ROC curve (AUROC or AUC—the area under the curve outlining the tradeoff between sensitivity and specificity across operating points). The primary metrics used to evaluate each model are overall accuracy and AUROC. Two validation methods were employed, including holdout set validation and K-Fold validation on the hold-out set. The hold-out set was established with a large 40% of the samples randomly selected in order to assist with reliability and reproducibility. Validation was performed on the holdout set, as well as with K-Fold validation on the holdout set with K = 5. Optuna hyperparameter tuning was completed with 50 runs. After completion of the above methods, a new version of df-analyze was released with support for an emerging deep learning method designed for tabular data, known as Gated Adaptive Network for Deep Automated Learning of Features (GANDALF) [44]. Df-analyze was re-accessed to assess this method as well (df-analyze access date: November 2024), and the experiments were re-run with GANDALF enabled. Due to the additional computational demands of GANDALF relative to the other machine learning methods assessed, df-analyze was run without redundancy-aware step-up feature selection enabled, as this was the slowest of our considered feature selection methods.

## 3. Results

### 3.1. Predicting Diagnosis

For predicting diagnosis when including US image features, the best-performing model was the random forest (RF) with embedded LGBM-based feature selection, achieving an accuracy of 98.1% and an AUROC of 99.3% across both validation methods, see Appendix B. The features that this model relied upon are outlined in Appendix C, which provides a ranking of their respective apparent importance to inform prediction.

When excluding the US image-based features, the best-performing model was LGBM with no (none) feature selection, achieving an accuracy of 80.1% and an AUROC of 87.3–88.0% across both validation methods, see Appendix D.

**Figure 1:**
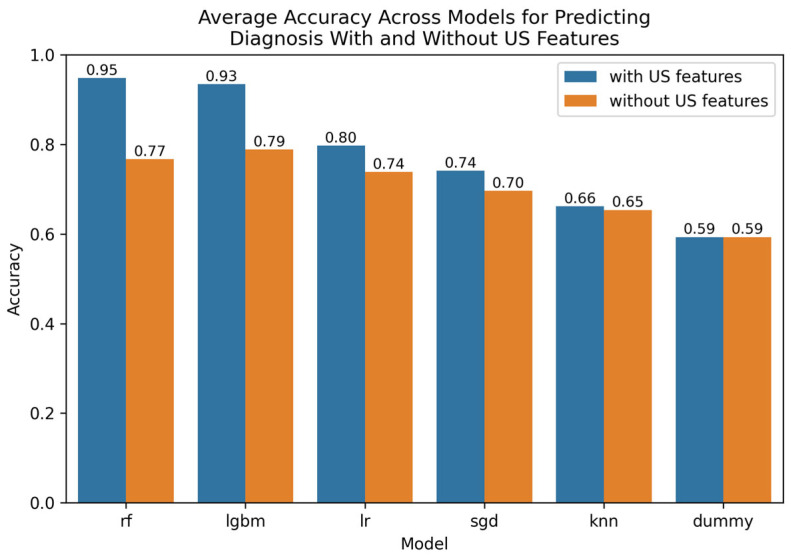
Comparative bar plot of leading models for predicting diagnosis with and without US features.

The Optuna hyperparameter-tuned model parameters for the leading techniques are provided in Appendix E. A comparative visualization of leading findings is provided in Figure 1.

### 3.2. Predicting Management

For predicting management, the best-performing models that included US-based image features were the random forest with association filter-based feature selection (assoc), achieving accuracies of 92.0–93.6% accuracy and an AUROC of 97.3–98.4% across both validation methods, see Appendix F. The association feature selection method selected for a large number of the available features in this dataset and is provided in detail in Appendix G. Note that a sorting of the importance of the features is provided both for numerical and categorical features, respectively. The leading features informing prediction, according to the association filter-based method’s reliance on mutual information, were C-reactive protein, Alvarado score, the appendix diameter, white blood cell count, and neutrophil percentage for the numerical variables, and ipsilateral rebound tenderness, diagnosis, peritonitis, severity, and surrounding tissue reaction for the categorical variables.

When predicting management without the US-based image features, the best-performing model was the random forest (RF) with no (none) feature selection, achieving accuracies of 92.0–93.9% and an AUROC of 97.0–98.0% across both validation methods, see Appendix H. Noteworthy is that our emerging redundancy-aware step-up feature selection method (wrap), which is biased in favour of unusually small feature sets, achieved near equal accuracies of 92.0–92.7% and an AUROC of 94.2–96.0%, based on just 11 features, as outlined in Appendix I. The leading features relied upon were peritonitis, white blood cell count, body temperature, weight, severity, and C-reactive protein.

**Figure 2:**
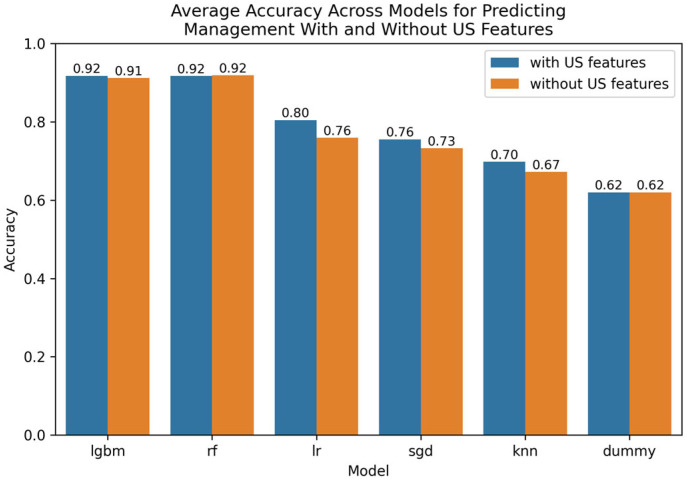
Comparative bar plot of leading models for predicting management with and without US features.

The Optuna hyperparameter-tuned model parameters for leading techniques are provided in Appendix E. A visualization of leading findings is provided in Figure 2.

### 3.3. Predicting Severity

For predicting severity, with US image features included, the best-performing model was logistic regression (LR) with wrapper-based redundancy-aware step-up feature selection (wrap), which achieved accuracy of 89.1–89.5% and an AUROC of 82.0–83.4% across both validation methods, see Appendix J. The feature selection results are provided in Appendix K. Leading features were meteorism (excess gas in the digestive tract), dysuria, weight, lower right abdominal pain, and free fluids.

When predicting severity with US image features excluded, the best-performing model was LGBM with filter-based association (assoc) feature selection, achieving an accuracy of 89.2–90.1% and an AUROC of 89.6–93.1% across both validation methods, see Appendix L. As is common, the association-based feature selection method selects a large number of the available features in this dataset. Also of interest, redundancy-aware step-up feature selection (wrap) produced similar results, achieving an accuracy of 88.8% and an AUROC of 80.5–81.1% when combined with logistic regression based on just five features, as outlined in Appendix M. The five features included were peritonitis, coughing pain, body temperature, thrombocyte count, and C-reactive protein.

**Figure 3:**
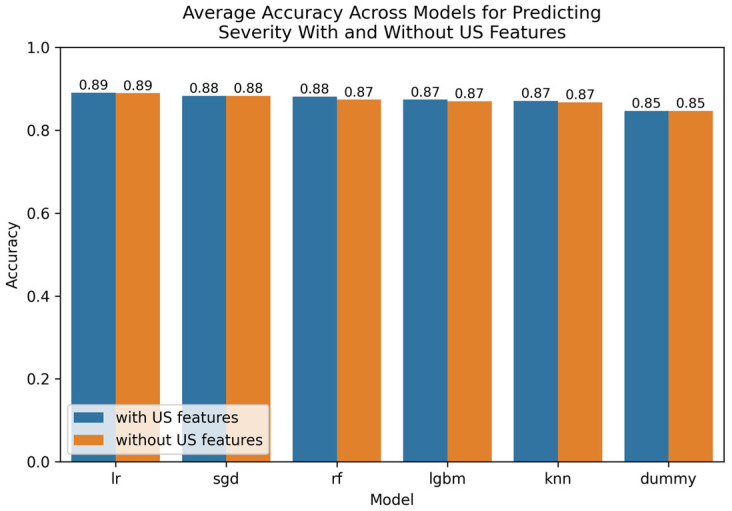
Comparative bar plot of leading models for predicting severity with and without US features.

The Optuna hyperparameter-tuned model parameters for leading techniques are provided in Appendix E. A visualization of leading findings is provided in Figure 3.

### 3.4. GANDALF Results

GANDALF [44] was run with an updated version of df-analyze, and so the results presented can only be roughly compared with the findings presented above due to it being run as an additional round of validation with unique randomization. When predicting diagnosis, the leading accuracy/AUROC for GANDALF was 80.5/90.6% with US features (filter-based prediction feature selection), and 66.7/75.7% without US features (filter-based prediction feature selection). When predicting management, the leading accuracy/AUROC for GANDALF was 91.5/96.9% with US features (no feature selection), and 90.5/97.5% without US features (embedded linear feature selection). When predicting severity, the leading accuracy/AUROC for GANDALF was 81.1/77.7% with US features (filter-based prediction feature selection), and 85.4/81.1% without US features (embedded linear feature selection).

## 4. Discussion

We performed a detailed study comparing several machine learning algorithms combined exhaustively with a variety of feature selection approaches applied to pediatric appendicitis diagnostics, management (treatment prediction), and severity. Results demonstrate that we are able to create high-performing models for each of the three main predictive tasks addressed. Our extensive use of feature selection has provided a variety of feature sets predictive of our three addressed target variables, information that can potentially assist in the clinical management of appendicitis and may inform the development of future AI technologies in this domain.

### 4.1. Interactions Between Machine Learning and Feature Selection Technologies

Our df-analyze benchmarking software has been previously used to assess machine learning and feature selection combinations that produce high-quality AI models to assist in schizophrenia diagnostics [45], chronic kidney disease diagnosis [46], mitigating bias in traffic stop outcomes [47], and studying proteins potentially linked with learning in the cerebral cortex [48]. In this study, we investigated the tool’s potential for use in three applications of pediatric appendicitis.

Logistic regression (LR) and stochastic gradient descent (SGD) were only among our top performers when using a feature selection method, suggesting that those methods are sensitive to being negatively biased from the inclusion of noisy, useless, and/or redundant features. In contrast, the light gradient boosting machine (LGBM) and the random forest (RF) models often performed well in predicting appendicitis diagnosis, management, and severity with and without feature selection methods. These results imply that the LGBM and RF are strong at ignoring noisy, useless, and/or redundant features in this application. These observations are expected as the LGBM and RF are both based on collections of decision tree classifiers, which are inherently capable of ignoring weak features, as they strongly tend not to be selected for in the splitting process that creates decisions at each split in each base learner decision tree. Our results also demonstrate potential from our novel redundancy-aware feature selection (FS) method, contributing to high-performing models in both management and severity prediction, based on relatively small feature sets. Such solutions have the potential to improve the explainability of our AI technologies through a greatly reduced feature set size. For management, our redundancy-aware FS method identified 11 features (see Appendix I), with the leading features relied upon being peritonitis, white blood cell count, body temperature, weight, severity, and C-reactive protein. For severity, our redundancy-aware FS method identified five features (see Appendix M): peritonitis, coughing pain, body temperature, thrombocyte count, and C-reactive protein. These feature sets are highly predictive of management and severity, respectively, and so may represent useful information for clinicians responsible for patient management.

### 4.2. Discussion of GANDALF Results

GANDALF [44], an emerging deep learning architecture designed for tabular data, upon which deep learners have traditionally been underperformers, was assessed as an addendum to this study. Results demonstrate overall good performance from GANDALF; however, it was not the leading AI technology in our trials in terms of predictive accuracy. That said, GANDALF was very competitive in predicting management and severity, especially in terms of AUROC scores, implying the method is capable of creating internal embeddings of feature representations that assist in delineating between our target classes of interest as assessed by AUROC. It is well known that deep learners in particular benefit from large sample sizes to train upon, and so it is expected that in this application, with relatively few samples compared with many other machine learning studies, GANDALF is disadvantaged.

### 4.3. Predictive Significance of US Image Features

For predicting diagnosis, the performance tables in Appendix H and Appendix J consistently show a decrease in predictive accuracy of our top-performing models of 10–20% in both performance on holdout set and 5-fold cross-validation on the holdout set when withholding US image features. The significant drop in performance suggests information in the US image features is important for diagnosing appendicitis and contributes to a mitigation in how biassed the resultant models are from predicting ground-truth diagnoses. When predicting management, there is no drop in performance across our top-performing models when US image features are removed (see Appendix C and Appendix L). Similar findings were observed in comparative performance when US image features are included/excluded when predicting Severity (see Appendix G and Appendix I). These results suggest US image-derived features are either not useful in predicting the management and severity of pediatric appendicitis or are redundant to non-US-based features available in this dataset.

### 4.4. Literature Comparison

The appendicitis dataset relied upon has been updated since the earliest publications focused on this work [35,37], supporting a more statistically powered analysis with 781 patients in our study, as opposed to 430 patients [35] and 579 patients [37]. Thus, any comparisons between our findings and the foundational papers on this dataset in the literature [35,37] are not exact comparisons due to the dataset size, as well as inevitably employing different validation strategies. Having more samples in the total dataset is expected to help improve predictive accuracies, as more samples are available for training, which is well known to improve the performance of machine learning models generally. Also noteworthy, our validation approach involved reserving 40% of the samples in the dataset for our hold-out testing to help ensure reliability. This has the potential to reduce our reported predictive accuracy, as only 60% of the total samples were available for training in a relatively small dataset. Previous work on this dataset employed validation with 10% of samples included in the testing pools [35]. Results indicate that our leading models produced AUROC scores of 0.993 for predicting diagnosis, 0.973–0.984 for predicting management, and 0.896–0.931 for predicting severity across our two validation methods. This compares favourably with literature work on a subset of this dataset [35], which reported AUROC scores of 0.96 (+/−0.01) for predicting diagnosis, and 0.94 (+/−0.02) for predicting management; however, our findings were approximately the same for predicting severity, with the literature reporting AUROC scores of 0.91 (+/−0.07) [35]. Our results are roughly in line with those from the literature [35], with some noteworthy improvements in AUROC scores in predicting diagnosis and management. The improved performance of our models may be attributable to the increased sample size available in our dataset, and features of df-analyze, such as Optuna hyperparameter tuning, extensive feature selection techniques evaluated, using state-of-the-art scikit learn implementations of learning machines in Python (as opposed to relying on R), and consideration of lightweight high-performing algorithms such as the light gradient boosting machine (LGBM), and LGBM-based embedded feature selection. It should also be noted that two additional studies have been conducted on the updated dataset used in this analysis, focused exclusively on diagnosis [38,39]. This includes an approach achieving 94.5% accuracy with the random forest [38], and an approach based on the Hybrid Bat algorithm achieving 94% accuracy [39]. An additional study was based on recursive feature elimination and the random forest, which did not report overall accuracies [39], but reported AUROC scores for diagnosis of 0.96 +/−0.02 [40]. In contrast, our approach, enhanced by Optuna hyperparameter tuning and feature selection, compares favourably with 98.1% accuracy and AUROC scores of 0.993 for diagnosis.

### 4.5. Future Work and Limitations

An interesting consideration that has resulted from this study relates to interactions between the target variables. There is potential value, for instance, in predicting diagnosis with and without knowledge of management, or predicting management with or without knowledge of the diagnosis. For instance, diagnosis is often not established until after surgical management, so the method selected for surgical management can potentially be a useful informative feature assisting in the predictive capacity of diagnosis. Conversely, management may benefit from knowledge of the final diagnosis if it is available. However, in situations where it is not (the patient’s final diagnosis is unknown), but the patient is proceeding to management/surgery, then a management prediction algorithm should not be informed as to the patient’s diagnosis when creating a technology to be relied upon clinically. Confounding issues, such as these, are important when creating a series of technologies to be relied upon for aiding clinical management of patients. Models can be created with and without knowledge of the other target variables of interest; thus, appropriate AI models can theoretically be relied upon clinically based on the availability (or not) of given target variables that may be helpful in informing prediction. Furthermore, AI technologies can be created that input a prediction of a target variable assessed by a different AI model. While this study is a research endeavour, and the models developed have not been clinically deployed, it is important for AI developers in medical applications to appreciate the various trade-offs and varying clinical utility of nearly identical models trained on almost the same set of potential predictor variables. Preliminary experiments indicate that high-performing models can be built with df-analyze for these applications with and without the inclusion of alternate target variables as features informing prediction. Limitations include that this study was performed on a single dataset, as this is the only dataset of its type publicly available; thus, independent dataset validation was not possible. Future work should involve validation on additional independent datasets in different healthcare environments to assess their generalisability across diverse pediatric populations. Future work should also involve consideration of emerging learning algorithms, such as updates to deep learners focused on tabular data.

## 5. Conclusions

We investigated the use of several machine learning technologies exhaustively combined with a variety of feature selection algorithms for predicting the diagnosis, management, and severity of pediatric appendicitis, with and without the inclusion of ultrasound image-derived features. Ultrasound image features were found to be important for maximizing accuracy when performing diagnostics, providing support for the value of imaging features in mitigating bias in the AI model relative to ground-truth diagnoses. However, findings imply that image-derived features are not as useful when predicting the management and severity of the condition. A variety of leading learning machines were presented based on variable subsets of the features identified by our redundancy-aware FS, providing detailed information that can potentially aid in the explainability of our AI models. The methods outlined in this study produced AI technologies with robust predictive potential in three applications focused on pediatric appendicitis as assessed by the area under the receiver operating characteristic curve. The technologies developed in this study could potentially help identify and manage young patients with suspected appendicitis. Advantages of the approach taken in this study include the consideration of a novel redundancy-aware step-up feature selection algorithm, consideration of an emerging deep learner optimized for tabular data (Gandalf), assessment of the value of US-derived features, and the creation of highly accurate AI models for three applications. Disadvantages include that this study did not consider convolutional neural networks that process the US images available in this dataset, as well as being reliant on a single dataset for all analyses. Future work will investigate the role of image analysis deep learners, including on additional datasets.

## Figures and Tables

**Table 1:** Diagnosis target variable.

	Appendicitis	No Appendicitis
Frequency	463	317
Proportion	463/780	317/780

**Table 2:** Management target variable.

	Conservative	Primary Surgical	Secondary Surgical	Simultaneous Appendectomy
Frequency	483	270	27	1
Proportion	483/781	270/781	27/781	1/781
Relative Frequency	61.84%	34.57%	3.46%	0.13%

**Table 3:** Severity target variable.

	Uncomplicated	Complicated
Frequency	662	119
Proportion	662/781	119/781
Relative Frequency	84.76%	15.24%

**Table 4:** Numeric feature statistics for patients with and without appendicitis.

Variable	Appendicitis: Mean, SD	No Appendicitis: Mean, SD
Age	11.08, 3.56	11.72, 3.46
BMI	18.45, 4.16	19.56, 4.62
Height	146.93, 20.43	149.51, 18.64
Weight	41.72, 17.47	45.25, 17.11
Length_of_Stay	5.11, 2.98	3.09, 0.98
Alvarado_Score	6.67, 1.93	4.83, 2.0
Paedriatic_Appendicitis_Score	5.82, 1.85	4.42, 1.81
Appendix_Diameter	8.7, 2.18	5.04, 1.17
Body_Temperature	37.52, 0.81	37.24, 1.0
WBC_Count	14.28, 5.34	10.33, 4.48
Neutrophil_Percentage	76.03, 12.63	65.6, 14.76
Segmented_Neutrophils	71.6, 12.51	55.23, 13.29
RBC_Count	4.79, 0.37	4.82, 0.64
Hemoglobin	13.38, 1.61	13.38, 1.02
RDW	13.4, 5.86	12.87, 0.87
Thrombocyte_Count	285.79, 70.83	284.48, 74.92
Ketones_in_Urine	1.15, 1.28	0.69, 1.11
RBC_in_Urine	0.4, 0.82	0.32, 0.71
WBC_in_Urine	0.24, 0.63	0.19, 0.55
CRP	44.9, 68.51	11.72, 24.92

## Data Availability

The dataset used in this study is publicly available and can be accessed at https://archive.ics.uci.edu/dataset/938/regensburg+pediatric+appendicitis (accessed on 30 September 2024). No new data were created or collected specifically for this study. Since this was a retrospective analysis of public domain data, no institutional review board approval was necessary for conducting this study.

## Abbreviations

The following abbreviations are used in this manuscript:

LGBM	Light Gradient Boosting Machine
RF	Random Forest
LR	Linear Regression
SGD	Stochastic Gradient Descent
AUROC	Area Under the Receiver Operating Characteristic Curve
US	Ultrasound
GANDALF	Gated Adaptive Network for Deep Automated Learning of Features

## Appendix A

Categorical feature statistics for each of three applications.

**Table A1:** Categorical feature statistics for the target variable Diagnosis.

Feature	Class	% Appendicitis	% No Appendicitis	% of Total
Sex	female	53.19	46.81	48.33
	male	65.01	34.99	51.67
Management	conservative	35.2	64.8	61.84
	primary surgical	99.26	0.74	34.57
	secondary surgical	96.15	3.85	3.46
	simultaneous appendectomy	0	100	0.13
Severity	complicated	99.16	0.84	15.24
	uncomplicated	52.19	47.81	84.76
Appendix on the US	no	30.04	69.96	35.14
	yes	75	25	64.86
Migratory Pain	no	56.23	43.77	72.7
	yes	66.82	33.18	27.3
Lower Right Abd Pain	no	36.59	63.41	5.3
	yes	60.44	39.56	94.7
Contralateral Rebound Tenderness	no	51.6	48.4	61.15
	yes	70.13	29.87	38.85
Coughing Pain	no	55.84	44.16	71.54
	yes	66.06	33.94	28.46
Nausea	no	48.91	51.09	41.47
	yes	66.45	33.55	58.53
Loss of Appetite	no	51.05	48.95	49.22
	yes	66.84	33.16	50.78
Neutrophilia	no	44.47	55.53	50.68
	yes	74.52	25.48	49.32
Dysuria	no	58.67	41.33	94.16
	yes	47.73	52.27	5.84
Stool	constipation	59.77	40.23	11.37
	constipation, diarrhea	100	0	0.13
	diarrhea	65.62	34.38	16.73
	normal	57.19	42.81	71.76
Peritonitis	generalized	87.8	12.2	5.3
	local	86.98	13.02	24.84
	no	47.04	52.96	69.86
Psoas Sign	no	60.67	39.33	68.59
	yes	52.56	47.44	31.41
Ipsilateral Rebound Tenderness	no	47.68	52.32	93.86
	yes	73.68	26.32	6.14
US_Performed	no	71.43	28.57	1.93
	yes	59.11	40.89	98.07
Free_Fluids	no	50.61	49.39	56.88
	yes	71.94	28.06	43.12
Appendix Wall Layers	intact	77.27	22.73	60.55
	partially raised	100	0	4.13
	raised	96.05	3.95	34.86
	upset	100	0	0.46
Target Sign	no	49.02	50.98	36.96
	yes	94.25	5.75	63.04
Appendicolith	no	90.91	9.09	47.83
	suspected	100	0	4.35
	yes	100	0	47.83
Perfusion	hyperperfused	96.77	3.23	49.21
	hypoperfused	96.43	3.57	44.44
	no	100	0	4.76
	present	100	0	1.59
Perforation	no	88.24	11.76	41.98
	not excluded	100	0	18.52
	suspected	66.67	33.33	3.7
	yes	100	0	35.8
Surrounding Tissue Reaction	no	63.64	36.36	17.46
	yes	94.23	5.77	82.54
Appendicular Abscess	no	86.15	13.85	76.47
	suspected	100	0	1.18
	yes	100	0	22.35
Pathological Lymph Nodes	no	59.18	40.82	24.14
	yes	53.25	46.75	75.86
Bowel Wall Thickening	no	50	50	44.44
	yes	85.45	14.55	55.56
Conglomerate of Bowel Loops	no	81.82	18.18	51.16
	yes	90.48	9.52	48.84
Ileus	no	83.78	16.22	61.67
	yes	100	0	38.33
Coprostasis	no	100	0	35.21
	yes	50	50	64.79
Meteorism	no	100	0	7.86
	yes	45.74	54.26	92.14
Enteritis	no	86.67	13.33	22.73
	yes	31.37	68.63	77.27

**Table A2:** Categorical feature statistics for the target variable management.

Feature	Class	Conservative	Primary Surgical	Secondary Surgical
Sex	female	65.52	29.97	4.24
	male	58.56	38.96	2.48
Severity	complicated	0	96.64	3.36
	uncomplicated	72.96	23.41	3.47
Diagnosis	appendicitis	36.72	57.88	5.4
	no appendicitis	98.74	0.63	0.32
Appendix_on_US	no	68.86	26.74	4.03
	yes	58.53	38.89	2.58
Migratory_Pain	no	63.17	33.81	2.85
	yes	60.66	36.02	3.32
Lower_Right_Abd_Pain	no	73.17	24.39	2.44
	yes	61.8	35.06	3
Contralateral_Rebound_Tenderness	no	70.79	27.29	1.71
	yes	50.67	44.63	4.7
Coughing_Pain	no	64.6	32.85	2.55
	yes	59.17	38.07	2.29
Nausea	no	73.83	23.36	2.8
	yes	54.3	42.6	2.87
Loss_of_Appetite	no	71.05	27.63	1.32
	yes	54.34	41.33	4.08
Neutrophilia	no	79.51	17.52	2.96
	yes	46.26	50.69	2.77
Dysuria	no	64.32	32.58	2.96
	yes	61.36	36.36	2.27
Stool	constipation	63.22	35.63	1.15
	constipation, diarrhea	0	100	0
	diarrhea	57.81	39.06	3.12
	normal	64.48	32.24	3.1
Peritonitis	generalized	14.63	82.93	2.44
	local	19.79	74.48	5.21
	no	81.3	16.67	2.04
Psoas_Sign	no	63.8	34.05	2.15
	yes	66.24	29.49	4.27
Ipsilateral_Rebound_Tenderness	no	80.03	18.76	1.2
	yes	47.37	50	2.63
US_Performed	no	26.67	46.67	26.67
	yes	62.78	34.34	2.75
Free_Fluids	no	74.57	22.49	2.93
	yes	46.45	50.32	2.9
Appendix_Wall_Layers	intact	71.97	26.52	1.52
	partially raised	0	100	0
	raised	17.11	76.32	6.58
	upset	0	100	0
Target_Sign	no	60.78	31.37	7.84
	yes	29.89	68.97	1.15
Appendicolith	no	54.55	36.36	9.09
	suspected	100	0	0
	yes	9.09	87.88	3.03
Perfusion	hyperperfused	48.39	45.16	6.45
	hypoperfused	14.29	78.57	7.14
	no	0	100	0
	present	0	100	0
Perforation	no	44.12	50	5.88
	not excluded	0	100	0
	suspected	66.67	33.33	0
	yes	0	100	0
Surrounding_Tissue_Reaction	no	77.27	22.73	0
	yes	26.44	69.71	3.85
Appendicular_Abscess	no	38.46	58.46	3.08
	suspected	0	100	0
	yes	0	89.47	10.53
Pathological_Lymph_Nodes	no	59.18	38.78	2.04
	yes	68.83	27.27	3.9
Bowel_Wall_Thickening	no	68.18	27.27	4.55
	yes	23.64	67.27	9.09
Conglomerate_of_Bowel_Loops	no	31.82	63.64	4.55
	yes	9.52	85.71	4.76
Ileus	no	27.03	62.16	8.11
	yes	0	95.65	4.35
Coprostasis	no	4	88	8
	yes	69.57	30.43	0
Meteorism	no	0	90.91	9.09
	yes	66.67	27.91	4.65
Enteritis	no	20	73.33	6.67
	yes	90.2	9.8	0

**Table A3:** Categorical feature statistics for target variable severity.

Feature	Class	Complicated	Uncomplicated	% of Total
Sex	female	14.85	85.15	48.33
	male	15.63	84.37	51.67
Management	conservative	0	100	61.84
	primary surgical	42.59	57.41	34.57
	secondary surgical	14.81	85.19	3.46
	simultaneous appendectomy	0	100	0.13
Diagnosis	appendicitis	25.49	74.51	59.36
	no appendicitis	0.32	99.68	40.64
Appendix_on_US	no	17.22	82.78	35.14
	yes	14.09	85.91	64.86
Migratory_Pain	no	15.12	84.88	72.7
	yes	15.17	84.83	27.3
Lower_Right_Abd_Pain	no	19.51	80.49	5.3
	yes	14.87	85.13	94.7
Contralateral_Rebound_Tenderness	no	11.73	88.27	61.15
	yes	19.46	80.54	38.85
Coughing_Pain	no	14.05	85.95	71.54
	yes	16.97	83.03	28.46
Nausea	no	5.61	94.39	41.47
	yes	21.85	78.15	58.53
Loss_of_Appetite	no	7.37	92.63	49.22
	yes	22.7	77.3	50.78
Neutrophilia	no	5.12	94.88	50.68
	yes	23.82	76.18	49.32
Dysuria	no	13.96	86.04	94.16
	yes	18.18	81.82	5.84
Stool	constipation	17.24	82.76	11.37
	constipation, diarrhea	100	0	0.13
	diarrhea	19.53	80.47	16.73
	normal	13.11	86.89	71.76
Peritonitis	generalized	51.22	48.78	5.3
	local	29.17	70.83	24.84
	no	7.22	92.78	69.86
Psoas_Sign	no	15.66	84.34	68.59
	yes	10.26	89.74	31.41
Ipsilateral_Rebound_Tenderness	no	6.54	93.46	93.86
	yes	23.68	76.32	6.14
US_Performed	no	13.33	86.67	1.93
	yes	15.07	84.93	98.07
Free_Fluids	no	7.58	92.42	56.88
	yes	23.55	76.45	43.12
Appendix_Wall_Layers	intact	5.3	94.7	60.55
	partially raised	66.67	33.33	4.13
	raised	32.89	67.11	34.86
	upset	100	0	0.46
Target_Sign	no	19.61	80.39	36.96
	yes	21.84	78.16	63.04
Appendicolith	no	9.09	90.91	47.83
	suspected	0	100	4.35
	yes	48.48	51.52	47.83
Perfusion	hyperperfused	16.13	83.87	49.21
	hypoperfused	28.57	71.43	44.44
	no	0	100	4.76
	present	100	0	1.59
Perforation	no	11.76	88.24	41.98
	not excluded	66.67	33.33	18.52
	suspected	33.33	66.67	3.7
	yes	68.97	31.03	35.8
Surrounding_Tissue_Reaction	no	6.82	93.18	17.46
	yes	30.29	69.71	82.54
Appendicular_Abscess	no	21.54	78.46	76.47
	suspected	100	0	1.18
	yes	78.95	21.05	22.35
Pathological_Lymph_Nodes	no	16.33	83.67	24.14
	yes	9.74	90.26	75.86
Bowel_Wall_Thickening	no	11.36	88.64	44.44
	yes	36.36	63.64	55.56
Conglomerate_of_Bowel_Loops	no	22.73	77.27	51.16
	yes	71.43	28.57	48.84
Ileus	no	10.81	89.19	61.67
	yes	82.61	17.39	38.33
Coprostasis	no	28	72	35.21
	yes	21.74	78.26	64.79
Meteorism	no	27.27	72.73	7.86
	yes	13.18	86.82	92.14
Enteritis	no	20	80	22.73
	yes	5.88	94.12	77.27

**Table A4:** Demographic/Other.

Variable	Variable Name in Data Files	Explanation	Mode and Time of Measurement	Variable Type and Values
Age, years	Age	Obtained from the date of birth	At hospital admission	Continuous
Sex	Sex	Registered gender	At hospital admission	Binary: female/male
Height, cm	Height	Patient’s height	At hospital admission	Continuous
Weight, kg	Weight	Patient’s weight	At hospital admission	Continuous
Body mass index (BMI), kg/m^2^	BMI	Measures body fat; patient’s weight divided by the square of the height	At hospital admission	Continuous
Length of stay, days	Length_of_Stay	Length of stay in the hospital	At discharge	Continuous

**Table A5:** Scoring.

Variable	Variable Name in Data Files	Explanation	Mode and Time of Measurement	Variable Type and Values
Alvarado score (AS), pts	Alvarado_Score	Patient’s score according to the scoring system	At hospital admission, after clinical examination and laboratory data	Discrete
Pediatric appendicitis score (PAS), pts	Pediatric_Appendicitis_Score	Patient’s score according to the scoring system	At hospital admission, after clinical examination and laboratory data	Discrete

**Table A6:** Clinical features.

Variable	Variable Name in Data Files	Explanation	Mode and Time of Measurement	Variable Type and Values
Peritonitis/ abdominal guarding	Peritonitis	Spasm of abdominal wall muscles detected on palpation, usually a result of inflammation	At hospital admission, during clinical examination, or after a few hours of observation, if needed, after analgesia	Categorical: no localized generalized
Migration of pain	Migratory_Pain	Abdominal pain; usually starts in the epigastrium and moves to the right lower quadrant	At hospital admission, during clinical examination or anamnesis	Binary: no/yes
Tenderness in right lower quadrant (RLQ)	Lower_Right_Abd_Pain	Right iliac fossa pain detected on palpation	At hospital admission, during clinical examination	Binary: no/yes
Contralateral rebound tnderness	Contralateral_Rebound_Tenderness	A state in which pain of the contralateral side (usually, the right lower quadrant) is felt on the release of pressure (usually, in the left lower quadrant) over the abdomen	At hospital admission, during clinical examination	Binary: no/yes
Ipsilateral rebound tenderness	Ipsilateral_Rebound_Tenderness	A state in which pain of the ipsilateral side is felt on the release of pressure over the abdomen	At hospital admission, during clinical examination	Binary: no/yes
Cough tenderness	Coughing_Pain	Abdominal pain from forced cough	At hospital admission, during clinical examination	Binary: no/yes
Psoas sign	Psoas_Sign	Abdominal pain produced by extension of the hip	At hospital admission, during clinical examination	Binary: negative/positive
Nausea/vomiting	Nausea	Feeling of sickness/ejection of contents from the stomach through the mouth	Anamnesis	Binary: no/yes
Anorexia	Loss_of_Appetite	Loss of appetite	Anamnesis	Binary: no/yes
Body temperature, °C	Body_Temperature	Measured by a thermometer placed in the rectum or in the auditory canal	At hospital admission or after a few hours of observation	Continuous
Dysuria	Dysuria	Pain or other difficulty during urination	Anamnesis	Binary: no/yes
Stool	Stool	Characteristics of bowel movements	Anamnesis	Categorical: · normal · diarrhea · obstipation

**Table A7:** Laboratory Features.

Variable	Variable Name in Data Files	Explanation	Mode and Time of Measurement	Variable Type and Values
White blood cell count (WBC), 10^3^/µL	WBC_Count	The number of leucocytes in a unit volume of blood; inflammation parameter	At hospital admission, obtained from a routine hemogram	Continuous
Red blood cell count (RBC), /pL	RBC_Count	The number of erythrocytes in a unit volume of bood	At hospital admission, obtained from a routine hemogram	Continuous
Hemoglobin, g/dL	Hemoglobin	Hemoglobin level; a red protein in the red blood cells that contains iron and is responsible for transporting oxygen	At hospital admission, obtained from a routine hemogram	Continuous
Red cell distribution width (RDW), %	RDW	A blood test that measures the differences in the volume and size of the erythrocytes	At hospital admission, obtained from a routine hemogram	Continuous
Thrombocyte count, /nL	Thrombocyte_Count	The number of platelets in a unit volume of bood	At hospital admission, obtained from a routine hemogram	Continuous
Neutrophils, %	Neutrophil_Percentage	Mature WBC in the granulocytic series	At hospital admission, obtained from differential WBC	Continuous
Neutrophilia, >= 75%	Neutrophilia	Relative neutrophilic leucocytosis, often a result of a bacterial infection	At hospital admission, obtained from differential WBC	Binary: no/yes
Segmented neutrophils, %	Segmented_Neutrophils	Most mature neutrophilic granulocytes present in circulating blood, increased during an inflammatory disorder	At hospital admission, obtained from differential WBC	Continuous
C-reactive protein (CRP), mg/L	CRP	Protein produced by the liver, elevated in case of inflammation, infection, or injury	At hospital admission, obtained from blood sample	Continuous
Ketones in urine	Ketones_in_Urine	Presence of ketone bodies in urine, e.g., in case of anorexia	At hospital admission, obtained from routine urine status	Categorical: o + ++ +++
Erythrocytes in urine	RBC_in_Urine	Blood in urine	At hospital admission, obtained from routine urine status	Categorical: neg: <5 ery/µL +: approx. 5–10 ery/µL ++: approx. 25 ery/µL +++: approx. 50 ery/µL
White blood cells in urine	WBC_in_Urine	Leucocytes in urine, e.g., in case of infection	At hospital admission, obtained from routine urine status	Categorical: no + ++ +++

**Table A8:** Ultrasound Features.

Variable	Variable Name in Data Files	Explanation	Mode and Time of Measurement	Variable Type and Values
Performed ultrasound (US)	US_Performed	If an abdominal ultrasonography was performed or not	At hospital admission, after clinical examination, or after a few hours of observation	Binary: no/yes
Visibility of appendix	Appendix_on_US	Detectability of the vermiform appendix during sonographic examination	At hospital admission, after clinical examination, or after a few hours of observation	Binary: no/yes
Appendix diameter, mm	Appendix_Diameter	Maximal outer diameter of the appendix	At hospital admission, after clinical examination, or after a few hours of observation	Continuous
Free intraperitoneal fluid	Free_Fluids	Free fluids inside the abdomen	At hospital admission, after clinical examination, or after a few hours of observation	Binary: no/yes
Appendix layer structure	Appendix_Wall_Layers	Distribution and characteristics of appendix layers, e.g., irregular in case of increasing inflammation	At hospital admission, after clinical examination, or after a few hours of observation	Binary: regular/irregular
Target sign	Target_Sign	Axial image of appendix with a fluid-filled centre surrounded by echogenic mucosa and submucosa and hypoechoic muscularis	At hospital admission, after clinical examination, or after a few hours of observation	Binary: no/yes
Appendix perfusion	Perfusion	Blood flow to the appendix wall	At hospital admission, after clinical examination, or after a few hours of observation	Categorical: unremarkable hypoperfused hyperperfused
Surrounding tissue reaction	Surrounding_Tissue_Reaction	Inflammation signs in tissue (i.a. in omentum/fat tissue) surrounding appendix	At hospital admission, after clinical examination, or after a few hours of observation	Binary: no/yes
Pathological lymph nodes	Pathological_Lymph_Nodes	Enlarged and inflamed intra-abdominal lymph nodes	At hospital admission, after clinical examination, or after a few hours of observation	Binary: no/yes
Location of pathological lymph nodes	Lymph_Node_Location	The location of pathological lymph nodes in the abdomen	At hospital admission, after clinical examination, or after a few hours of observation	Free-form text (in German)
Thickening of the bowel wall	Bowel_Wall_Thickening	Edema of the intestinal wall, >2–3 mm for small bowel wall thickening	At hospital admission, after clinical examination, or after a few hours of observation	Binary: no/yes
Ileus	Ileus	Sonographic signs of paralytic ileus (e.g., dilated intestinal loops, pendulum peristalsis or absence of peristalsis)	At hospital admission, after clinical examination, or after a few hours of observation	Binary: no/yes
Coprostasis	Coprostasis	Fecal impaction in the colon	At hospital admission, after clinical examination, or after a few hours of observation	Binary: no/yes
Meteorism	Meteorism	Accumulation of gas in the intestine	At hospital admission. after clinical examination, or after a few hours of observation	Binary: no/yes
Enteritis	Enteritis	Sonographic features of gastroenteritis, e.g., wall thickening of the ileum, increased peristalsis	At hospital admission, after clinical examination, or after a few hours of observation	Binary: no/yes
Appendicolith	Apendicolith	Presence of fecalith in the appendix, e.g., acoustic shadow	At hospital admission, after clinical examination, or after a few hours of observation	Binary: no/yes
Perforation	Perforation	Signs of appendix perforation in US	At hospital admission, after clinical examination, or after a few hours of observation	Binary: no/yes
Appendicular abscess	Appendicular_Abscess	Appendiceal mass	At hospital admission, after clinical examination, or after a few hours of observation	Binary: no/yes
Location of abscess	Abscess_Location	Location of the abscess intraperitoneal	At hospital admission, after clinical examination, or after a few hours of observation	Free-form text (in German)
Conglomerate of bowel loops	Conglomerate_of_Bowel_Loops	Small intestine conglomerate as a sign of intraperitoneal inflammation	At hospital admission, after clinical examination, or after a few hours of observation	Binary: no/yes
Gynecological findings	Gynecological_Findings	Gynecological abnormalities, e.g., cysts, ovarian torsion	At hospital admission, after clinical examination, or after a few hours of observation	Free-form text (in German)
Ultrasound images	NA	Snapshots from the abdominal ultrasound exams	At hospital admission, after clinical examination, or after a few hours of observation	Images in BMP format

**Table A9:** Diagnosis/management/severity target variables.

Variable	Variable Name in Data Files	Explanation	Mode and Time of Measurement	Variable Type and Values
Presumptive diagnosis	Diagnosis_Presumptive	Patient’s suspected diagnosis	At hospital admission, after clinical examination, or after a few hours of observation	Free-form text (in German)
Diagnosis	Diagnosis	Patient’s diagnosis, histologically confirmed for operated patients. Conservatively managed patients were labelled as having appendicitis if they had an AS or PAS of ≥ 4 and an appendix diameter of ≥6 mm	At hospital admission, after clinical examination, or after a few hours of observation	Binary: no appendicitis/appendicitis
Management	Management	Management of the patient assigned by a senior pediatric surgeon: operative (appendectomy: laparoscopic, open or conversion) or conservative (without antibiotics). In case of the secondary surgery after prior stay, the patient was labelled as operatively managed.	At hospital admission after clinical examination, or after a few hours of observation; or during follow-up.	Categorical: conservative primary surgical secondary surgical
Severity	Severity	Severity of appendicitis: uncomplicated: subacute/catharral, fibrosis; phlegmonous or complicated: gangrenous, perforated, abscessed	At hospital admission after clinical examination, or after a few hours of observation; or during follow-up.	Binary: uncomplicated or no appendicitis/complicated appendicitis

## Appendix B

Results from predicting Diagnosis with US image features.

**Table A10:** Diagnosis with US image features holdout set performance.

Model	Selection	Embed_Selector	Acc	Auroc	Bal-Acc	F1	Npv	Ppv	Sens	Spec
rf	embed_lgbm	lgbm	0.981	0.993	0.978	0.980	0.992	0.974	0.978	0.961
lgbm	embed_linear	linear	0.981	0.993	0.979	0.980	0.984	0.979	0.979	0.969
rf	pred	none	0.981	0.993	0.979	0.980	0.984	0.979	0.979	0.969
lgbm	none	none	0.981	0.994	0.979	0.980	0.984	0.979	0.979	0.969
lgbm	pred	none	0.978	0.996	0.976	0.977	0.976	0.978	0.976	0.969
rf	embed_linear	linear	0.978	0.991	0.974	0.977	0.992	0.968	0.974	0.953
lgbm	assoc	none	0.978	0.994	0.975	0.977	0.984	0.973	0.975	0.961
lgbm	embed_lgbm	lgbm	0.978	0.996	0.976	0.977	0.976	0.978	0.976	0.969
rf	none	none	0.965	0.992	0.958	0.963	0.992	0.948	0.958	0.921
rf	assoc	none	0.952	0.993	0.942	0.949	0.991	0.929	0.942	0.890
rf	wrap	none	0.875	0.945	0.867	0.870	0.861	0.884	0.867	0.827
lr	pred	none	0.865	0.947	0.864	0.862	0.820	0.899	0.864	0.858
lgbm	wrap	none	0.862	0.950	0.857	0.857	0.833	0.882	0.857	0.827
sgd	pred	none	0.837	0.843	0.823	0.828	0.833	0.838	0.823	0.748
lr	assoc	none	0.833	0.910	0.827	0.827	0.795	0.859	0.827	0.795
lr	none	none	0.833	0.910	0.827	0.827	0.795	0.859	0.827	0.795
lr	embed_linear	linear	0.833	0.910	0.827	0.827	0.795	0.859	0.827	0.795
lr	embed_lgbm	lgbm	0.804	0.893	0.794	0.796	0.770	0.826	0.794	0.740
sgd	embed_linear	linear	0.779	0.798	0.759	0.765	0.769	0.784	0.759	0.654
sgd	none	none	0.766	0.753	0.753	0.755	0.725	0.792	0.753	0.685
sgd	assoc	none	0.760	0.748	0.748	0.749	0.713	0.789	0.748	0.685
sgd	wrap	none	0.760	0.795	0.753	0.752	0.700	0.802	0.753	0.717
lr	wrap	none	0.753	0.847	0.724	0.730	0.766	0.748	0.724	0.567
sgd	embed_lgbm	lgbm	0.753	0.743	0.743	0.743	0.702	0.787	0.743	0.685
knn	none	none	0.683	0.715	0.651	0.653	0.649	0.697	0.651	0.480
knn	embed_linear	linear	0.683	0.721	0.644	0.644	0.671	0.687	0.644	0.433
knn	assoc	none	0.683	0.721	0.644	0.644	0.671	0.687	0.644	0.433
knn	pred	none	0.676	0.722	0.634	0.633	0.667	0.679	0.634	0.409
knn	embed_lgbm	lgbm	0.657	0.677	0.627	0.628	0.602	0.682	0.627	0.465
knn	wrap	none	0.622	0.601	0.601	0.602	0.539	0.670	0.601	0.488
dummy	embed_lgbm	lgbm	0.593	0.500	0.500	0.372	nan	0.593	0.500	0.000
dummy	wrap	none	0.593	0.500	0.500	0.372	nan	0.593	0.500	0.000
dummy	pred	none	0.593	0.500	0.500	0.372	nan	0.593	0.500	0.000
dummy	none	none	0.593	0.500	0.500	0.372	nan	0.593	0.500	0.000
dummy	embed_linear	linear	0.593	0.500	0.500	0.372	nan	0.593	0.500	0.000
dummy	assoc	none	0.593	0.500	0.500	0.372	nan	0.593	0.500	0.000

nan = Not a Number.

**Table A11:** Diagnosis with US image features 5-fold performance on the holdout set.

Model	Selection	Embed_Selector	Acc	Auroc	Bal-Acc	F1	Npv	Ppv	Sens	Spec
rf	embed_linear	linear	0.984	0.993	0.981	0.983	0.992	0.979	0.981	0.968
lgbm	assoc	none	0.984	0.995	0.983	0.983	0.985	0.984	0.983	0.976
lgbm	pred	none	0.981	0.997	0.980	0.980	0.977	0.984	0.980	0.976
lgbm	none	none	0.981	0.997	0.977	0.980	0.992	0.974	0.977	0.960
rf	embed_lgbm	lgbm	0.981	0.993	0.977	0.980	0.992	0.974	0.977	0.960
rf	pred	none	0.978	0.994	0.975	0.977	0.985	0.974	0.975	0.960
lgbm	embed_linear	linear	0.974	0.993	0.971	0.973	0.985	0.969	0.971	0.952
rf	none	none	0.962	0.992	0.956	0.960	0.977	0.954	0.956	0.929
rf	assoc	none	0.926	0.988	0.932	0.925	0.880	0.971	0.932	0.960
lgbm	wrap	none	0.859	0.944	0.851	0.851	0.848	0.881	0.851	0.810
rf	wrap	none	0.859	0.949	0.849	0.852	0.853	0.868	0.849	0.794
lr	pred	none	0.856	0.939	0.853	0.851	0.810	0.891	0.853	0.842
lgbm	embed_lgbm	lgbm	0.827	0.909	0.819	0.819	0.792	0.853	0.819	0.778
lr	embed_linear	linear	0.811	0.900	0.805	0.803	0.763	0.851	0.805	0.778
lr	none	none	0.811	0.899	0.805	0.803	0.763	0.851	0.805	0.778
lr	assoc	none	0.811	0.899	0.805	0.803	0.763	0.851	0.805	0.778
sgd	pred	none	0.792	0.801	0.785	0.784	0.737	0.832	0.785	0.755
lr	embed_lgbm	lgbm	0.785	0.873	0.774	0.773	0.746	0.817	0.774	0.715
sgd	embed_linear	linear	0.750	0.806	0.747	0.743	0.678	0.806	0.747	0.731
sgd	assoc	none	0.750	0.743	0.743	0.741	0.685	0.798	0.743	0.707
sgd	none	none	0.743	0.741	0.737	0.734	0.674	0.799	0.737	0.707
sgd	embed_lgbm	lgbm	0.731	0.726	0.726	0.723	0.659	0.787	0.726	0.700
lr	wrap	none	0.712	0.809	0.686	0.688	0.685	0.728	0.686	0.550
knn	embed_linear	linear	0.683	0.719	0.641	0.640	0.683	0.684	0.641	0.417
knn	assoc	none	0.683	0.719	0.641	0.640	0.683	0.684	0.641	0.417
sgd	wrap	none	0.682	0.684	0.675	0.672	0.605	0.744	0.675	0.636
knn	pred	none	0.679	0.725	0.645	0.643	0.641	0.695	0.645	0.462
knn	none	none	0.676	0.739	0.646	0.649	0.639	0.696	0.646	0.487
knn	embed_lgbm	lgbm	0.670	0.717	0.643	0.642	0.606	0.701	0.643	0.503
dummy	embed_lgbm	lgbm	0.593	0.500	0.500	0.372	nan	0.593	0.500	0.000
dummy	wrap	none	0.593	0.500	0.500	0.372	nan	0.593	0.500	0.000
dummy	pred	none	0.593	0.500	0.500	0.372	nan	0.593	0.500	0.000
dummy	none	none	0.593	0.500	0.500	0.372	nan	0.593	0.500	0.000
dummy	embed_linear	linear	0.593	0.500	0.500	0.372	nan	0.593	0.500	0.000
dummy	assoc	none	0.593	0.500	0.500	0.372	nan	0.593	0.500	0.000
knn	wrap	none	0.580	0.561	0.561	0.560	0.484	0.643	0.561	0.463

nan = Not a Number.

## Appendix C

Feature selection results for leading wrapper-based embedded LGBM feature selection for predicting Diagnosis with US image features.

**Table A12:** Selection scores (Importances: Larger magnitude = More important).

Feature	Score
Management_surgical	6.800 × 10^1^
Appendix_Diameter	5.800 × 10^1^
Appendix_Diameter_NAN	4.900 × 10^1^
Thrombocyte_Count	3.400 × 10^1^
Age	3.400 × 10^1^
Paedriatic_Appendicitis_Score	2.900 × 10^1^
WBC_Count	2.700 × 10^1^
Alvarado_Score	2.500 × 10^1^
CRP	2.200 × 10^1^
Appendix_on_US_yes	1.800 × 10^1^
Hemoglobin	1.400 × 10^1^
RDW	1.400 × 10^1^
Neutrophil_Percentage	1.300 × 10^1^
BMI	1.000 × 10^1^
Body_Temperature	9.000 × 10^0^
RBC_Count	8.000 × 10^0^
Coughing_Pain_yes	7.000 × 10^0^
Height	4.000 × 10^0^
Surrounding_Tissue_Reaction_nan	2.000 × 10^0^
Peritonitis_no	2.000 × 10^0^
Weight	1.000 × 10^0^
Contralateral_Rebound_Tenderness_yes	1.000 × 10^0^
Psoas_Sign_yes	1.000 × 10^0^

## Appendix D

Results from predicting diagnosis without US image features.

**Table A13:** Diagnosis without US image features holdout set performance.

Model	Selection	Embed_Selector	Acc	Auroc	Bal-Acc	F1	Npv	Ppv	Sens	Spec
lgbm	none	none	0.801	0.873	0.798	0.796	0.744	0.844	0.798	0.780
sgd	wrap	none	0.792	0.780	0.780	0.782	0.758	0.812	0.780	0.717
lgbm	assoc	none	0.782	0.882	0.778	0.776	0.722	0.827	0.778	0.756
rf	embed_lgbm	lgbm	0.779	0.864	0.778	0.774	0.710	0.833	0.778	0.772
lgbm	embed_lgbm	lgbm	0.776	0.871	0.766	0.767	0.728	0.807	0.766	0.717
rf	none	none	0.769	0.861	0.772	0.765	0.690	0.838	0.772	0.787
rf	embed_linear	linear	0.766	0.859	0.768	0.762	0.688	0.833	0.768	0.780
rf	assoc	none	0.766	0.861	0.751	0.754	0.733	0.786	0.751	0.669
rf	wrap	none	0.760	0.862	0.742	0.746	0.732	0.775	0.742	0.646
rf	pred	none	0.760	0.858	0.744	0.747	0.724	0.781	0.744	0.661
lgbm	embed_linear	linear	0.753	0.872	0.746	0.745	0.692	0.797	0.746	0.709
lr	wrap	none	0.750	0.823	0.730	0.734	0.725	0.764	0.730	0.622
lgbm	pred	none	0.747	0.855	0.735	0.736	0.697	0.779	0.735	0.669
lgbm	wrap	none	0.744	0.858	0.734	0.734	0.685	0.784	0.734	0.685
lr	pred	none	0.740	0.847	0.726	0.728	0.695	0.768	0.726	0.646
lr	embed_linear	linear	0.734	0.814	0.710	0.715	0.712	0.745	0.710	0.583
lr	assoc	none	0.728	0.815	0.704	0.708	0.702	0.740	0.704	0.575
sgd	embed_linear	linear	0.724	0.753	0.707	0.710	0.678	0.751	0.707	0.614
lr	none	none	0.715	0.809	0.692	0.695	0.679	0.733	0.692	0.567
sgd	pred	none	0.715	0.785	0.699	0.701	0.661	0.747	0.699	0.614
lr	embed_lgbm	lgbm	0.712	0.769	0.684	0.688	0.687	0.723	0.684	0.535
sgd	none	none	0.712	0.766	0.695	0.697	0.658	0.744	0.695	0.606
sgd	embed_lgbm	lgbm	0.708	0.689	0.689	0.691	0.661	0.735	0.689	0.583
sgd	assoc	none	0.705	0.763	0.675	0.678	0.684	0.714	0.675	0.512
knn	wrap	none	0.689	0.682	0.659	0.662	0.656	0.704	0.659	0.496
knn	none	none	0.686	0.713	0.654	0.656	0.656	0.699	0.654	0.480
knn	embed_lgbm	lgbm	0.673	0.721	0.633	0.632	0.654	0.680	0.633	0.417
knn	assoc	none	0.660	0.659	0.623	0.623	0.621	0.676	0.623	0.425
knn	pred	none	0.647	0.667	0.616	0.617	0.588	0.674	0.616	0.449
knn	embed_linear	linear	0.644	0.620	0.620	0.621	0.574	0.681	0.620	0.488
dummy	wrap	none	0.593	0.500	0.500	0.372	nan	0.593	0.500	0.000
dummy	embed_linear	linear	0.593	0.500	0.500	0.372	nan	0.593	0.500	0.000
dummy	none	none	0.593	0.500	0.500	0.372	nan	0.593	0.500	0.000
dummy	pred	none	0.593	0.500	0.500	0.372	nan	0.593	0.500	0.000
dummy	embed_lgbm	lgbm	0.593	0.500	0.500	0.372	nan	0.593	0.500	0.000
dummy	assoc	none	0.593	0.500	0.500	0.372	nan	0.593	0.500	0.000

nan = Not a Number.

**Table A14:** Diagnosis without US image features 5-fold performance on the holdout set.

Model	Selection	Embed_Selector	Acc	Auroc	Bal-Acc	F1	Npv	Ppv	Sens	Spec
lgbm	embed_lgbm	lgbm	0.808	0.878	0.805	0.802	0.756	0.855	0.805	0.794
lgbm	none	none	0.801	0.880	0.798	0.795	0.753	0.847	0.798	0.779
lgbm	embed_linear	linear	0.795	0.894	0.788	0.787	0.751	0.833	0.788	0.755
lgbm	assoc	none	0.785	0.883	0.784	0.780	0.720	0.840	0.784	0.779
rf	wrap	none	0.782	0.865	0.776	0.774	0.730	0.826	0.776	0.747
rf	pred	none	0.779	0.867	0.776	0.773	0.714	0.831	0.776	0.763
rf	assoc	none	0.772	0.870	0.765	0.764	0.715	0.816	0.765	0.731
lgbm	pred	none	0.772	0.864	0.763	0.762	0.720	0.809	0.763	0.715
lgbm	wrap	none	0.769	0.866	0.775	0.765	0.686	0.856	0.775	0.810
lr	pred	none	0.766	0.831	0.750	0.752	0.728	0.790	0.750	0.667
rf	none	none	0.763	0.853	0.748	0.751	0.728	0.785	0.748	0.670
rf	embed_linear	linear	0.759	0.866	0.766	0.755	0.671	0.851	0.766	0.802
rf	embed_lgbm	lgbm	0.747	0.861	0.741	0.739	0.692	0.794	0.741	0.709
lr	wrap	none	0.747	0.815	0.733	0.734	0.704	0.778	0.733	0.660
lr	assoc	none	0.743	0.800	0.729	0.730	0.696	0.773	0.729	0.652
lr	embed_linear	linear	0.737	0.799	0.722	0.723	0.687	0.768	0.722	0.644
lr	none	none	0.730	0.798	0.714	0.716	0.682	0.761	0.714	0.628
sgd	wrap	none	0.718	0.710	0.710	0.708	0.648	0.771	0.710	0.668
sgd	pred	none	0.718	0.775	0.698	0.700	0.667	0.746	0.698	0.596
lr	embed_lgbm	lgbm	0.708	0.754	0.686	0.686	0.657	0.738	0.686	0.573
sgd	assoc	none	0.695	0.759	0.677	0.676	0.636	0.735	0.677	0.580
sgd	none	none	0.692	0.750	0.678	0.676	0.626	0.741	0.678	0.604
knn	wrap	none	0.686	0.706	0.671	0.671	0.625	0.728	0.671	0.590
knn	pred	none	0.683	0.716	0.669	0.670	0.618	0.727	0.669	0.598
sgd	embed_lgbm	lgbm	0.679	0.670	0.670	0.668	0.602	0.738	0.670	0.621
sgd	embed_linear	linear	0.676	0.736	0.665	0.663	0.597	0.737	0.665	0.612
knn	none	none	0.670	0.735	0.639	0.640	0.634	0.689	0.639	0.472
knn	embed_lgbm	lgbm	0.657	0.722	0.619	0.617	0.613	0.673	0.619	0.416
knn	assoc	none	0.654	0.680	0.638	0.639	0.580	0.702	0.638	0.552
dummy	embed_lgbm	lgbm	0.593	0.500	0.500	0.372	nan	0.593	0.500	0.000
dummy	pred	none	0.593	0.500	0.500	0.372	nan	0.593	0.500	0.000
dummy	embed_linear	linear	0.593	0.500	0.500	0.372	nan	0.593	0.500	0.000
dummy	none	none	0.593	0.500	0.500	0.372	nan	0.593	0.500	0.000
dummy	wrap	none	0.593	0.500	0.500	0.372	nan	0.593	0.500	0.000
dummy	assoc	none	0.593	0.500	0.500	0.372	nan	0.593	0.500	0.000
knn	embed_linear	linear	0.570	0.569	0.557	0.556	0.474	0.641	0.557	0.487

nan = Not a Number.

## Appendix E

Hyperparameters established by Optuna hyperparameter tuning for each of our leading models.

**Table A15:** Optuna hyperparameters, for example, leading models.

Target	US Features	Model	Feature Selection	Hyperparameters
Diagnosis	Yes	rf	embed_linear	{‘verbosity’: −1, ‘boosting_type’: ‘rf’, ‘bagging_freq’: 1, ‘bagging_fraction’: 0.6424705933428012, ‘n_estimators’: 100, ‘reg_alpha’: 0.0003532789339921058, ‘reg_lambda’: 0.004369030571226374, ‘num_leaves’: 8, ‘colsample_bytree’: 0.8437223587619459, ‘subsample’: 0.403473633073295, ‘subsample_freq’: 1, ‘min_child_samples’: 5}
Diagnosis	No	lgbm	embed_lgbm	{‘verbosity’: −1, ‘n_estimators’: 50, ‘reg_alpha’: 0.06466023097198124, ‘reg_lambda’: 0.022294761212156983, ‘num_leaves’: 15, ‘colsample_bytree’: 0.5464250771120893, ‘subsample’: 0.5536293838457955, ‘subsample_freq’: 7, ‘min_child_samples’: 29}
Management	Yes	lgbm	assoc	{‘verbosity’: −1, ‘n_estimators’: 150, ‘reg_alpha’: 0.01918207182498792, ‘reg_lambda’: 7.461771397395436, ‘num_leaves’: 2,’colsample_bytree’: 0.5614712282427238, ‘subsample’: 0.8168115609573287, ‘subsample_freq’: 5, ‘min_child_samples’: 7}
Management	No	lgbm	no_select	{‘verbosity’: −1, ‘n_estimators’: 50, ‘reg_alpha’: 1.2550179156417959 × 10^−8^, ‘reg_lambda’: 1.9742923076305905 × 10^−8^, ‘num_leaves’: 2, ‘colsample_bytree’: 0.9856142911837322, ‘subsample’: 0.7805261984723494, ‘subsample_freq’: 0, ‘min_child_samples’: 5}
Severity	Yes	lr	wrap	{‘max_iter’: 2000, ‘penalty’: ‘elasticnet’, ‘solver’: ‘saga’, ‘l1_ratio’: 0.09092139813688659, ‘C’: 0.0007760418893874168}
Severity	No	lgbm	assoc	{‘verbosity’: −1, ‘n_estimators’: 200, ‘reg_alpha’: 0.025561180230324252, ‘reg_lambda’: 0.0020714646371430326, ‘num_leaves’: 67, ‘colsample_bytree’: 0.4887103613060258, ‘subsample’: 0.5044229983427804, ‘subsample_freq’: 3, ‘min_child_samples’: 18}

## Appendix F

Results from predicting management with US image features.

**Table A16:** Management with US image features holdout set performance.

Model	Selection	Embed_Selector	Acc	Auroc	Bal-Acc	F1	Npv	Ppv	Sens	Spec
rf	assoc	none	0.936	0.984	0.922	0.931	0.963	0.922	0.922	0.866
rf	embed_linear	linear	0.936	0.978	0.922	0.931	0.963	0.922	0.922	0.866
rf	none	none	0.933	0.980	0.917	0.927	0.971	0.914	0.917	0.849
lgbm	none	none	0.930	0.982	0.916	0.924	0.953	0.917	0.916	0.857
rf	pred	none	0.930	0.977	0.914	0.924	0.962	0.913	0.914	0.849
lgbm	pred	none	0.927	0.980	0.911	0.920	0.953	0.913	0.911	0.849
rf	wrap	none	0.923	0.946	0.902	0.916	0.980	0.897	0.902	0.815
rf	embed_lgbm	lgbm	0.923	0.980	0.909	0.917	0.944	0.913	0.909	0.849
lgbm	assoc	none	0.923	0.981	0.907	0.917	0.952	0.909	0.907	0.840
lgbm	embed_lgbm	lgbm	0.923	0.982	0.906	0.916	0.961	0.905	0.906	0.832
lgbm	embed_linear	linear	0.920	0.983	0.903	0.913	0.952	0.904	0.903	0.832
lgbm	wrap	none	0.920	0.943	0.900	0.912	0.970	0.897	0.900	0.815
lr	pred	none	0.879	0.949	0.855	0.866	0.909	0.864	0.855	0.756
lr	embed_linear	linear	0.866	0.929	0.838	0.851	0.905	0.849	0.838	0.723
lr	none	none	0.866	0.929	0.838	0.851	0.905	0.849	0.838	0.723
lr	assoc	none	0.863	0.924	0.834	0.847	0.904	0.845	0.834	0.714
lr	embed_lgbm	lgbm	0.853	0.920	0.823	0.836	0.892	0.836	0.823	0.697
sgd	pred	none	0.847	0.890	0.837	0.837	0.798	0.876	0.837	0.798
sgd	assoc	none	0.827	0.839	0.823	0.819	0.756	0.876	0.823	0.807
lr	wrap	none	0.827	0.897	0.784	0.801	0.911	0.799	0.784	0.605
sgd	none	none	0.805	0.789	0.789	0.791	0.754	0.834	0.789	0.723
sgd	embed_linear	linear	0.802	0.872	0.785	0.788	0.752	0.830	0.785	0.714
sgd	wrap	none	0.780	0.792	0.759	0.762	0.727	0.808	0.759	0.672
sgd	embed_lgbm	lgbm	0.776	0.852	0.746	0.754	0.747	0.790	0.746	0.622
knn	embed_lgbm	lgbm	0.741	0.772	0.699	0.706	0.721	0.749	0.699	0.521
knn	pred	none	0.719	0.779	0.656	0.659	0.746	0.712	0.656	0.395
knn	none	none	0.696	0.757	0.617	0.606	0.773	0.684	0.617	0.286
knn	embed_linear	linear	0.696	0.757	0.617	0.606	0.773	0.684	0.617	0.286
knn	assoc	none	0.696	0.757	0.617	0.606	0.773	0.684	0.617	0.286
knn	wrap	none	0.687	0.720	0.637	0.640	0.630	0.707	0.637	0.429
dummy	embed_lgbm	lgbm	0.620	0.500	0.500	0.383	nan	0.620	0.500	0.000
dummy	wrap	none	0.620	0.500	0.500	0.383	nan	0.620	0.500	0.000
dummy	pred	none	0.620	0.500	0.500	0.383	nan	0.620	0.500	0.000
dummy	none	none	0.620	0.500	0.500	0.383	nan	0.620	0.500	0.000
dummy	embed_linear	linear	0.620	0.500	0.500	0.383	nan	0.620	0.500	0.000
dummy	assoc	none	0.620	0.500	0.500	0.383	nan	0.620	0.500	0.000

nan = Not a Number.

**Table A17:** Management with US image features 5-fold performance on the holdout set.

Model	Selection	Embed_Selector	Acc	Auroc	Bal-Acc	F1	Npv	Ppv	Sens	Spec
lgbm	assoc	none	0.930	0.976	0.918	0.924	0.945	0.922	0.918	0.866
lgbm	embed_linear	linear	0.930	0.973	0.918	0.924	0.944	0.923	0.918	0.867
rf	embed_lgbm	lgbm	0.927	0.973	0.912	0.920	0.953	0.914	0.912	0.850
rf	none	none	0.920	0.968	0.907	0.913	0.937	0.914	0.907	0.850
rf	embed_linear	linear	0.920	0.971	0.905	0.913	0.944	0.909	0.905	0.841
rf	assoc	none	0.920	0.973	0.908	0.914	0.929	0.917	0.908	0.858
lgbm	embed_lgbm	lgbm	0.920	0.969	0.910	0.914	0.919	0.922	0.910	0.866
lgbm	wrap	none	0.911	0.937	0.893	0.902	0.942	0.897	0.893	0.816
rf	pred	none	0.911	0.968	0.896	0.902	0.928	0.905	0.896	0.833
lgbm	none	none	0.907	0.976	0.895	0.900	0.914	0.909	0.895	0.841
lgbm	pred	none	0.907	0.971	0.895	0.900	0.911	0.906	0.895	0.841
rf	wrap	none	0.904	0.938	0.886	0.895	0.936	0.892	0.886	0.808
lr	pred	none	0.882	0.938	0.864	0.871	0.890	0.881	0.864	0.790
lr	embed_linear	linear	0.805	0.892	0.778	0.786	0.795	0.812	0.778	0.664
lr	none	none	0.805	0.892	0.778	0.786	0.795	0.812	0.778	0.664
sgd	pred	none	0.801	0.861	0.789	0.789	0.748	0.839	0.789	0.739
lr	assoc	none	0.799	0.888	0.771	0.779	0.786	0.808	0.771	0.656
lr	embed_lgbm	lgbm	0.782	0.877	0.750	0.759	0.773	0.789	0.750	0.614
sgd	wrap	none	0.770	0.778	0.756	0.756	0.711	0.814	0.756	0.697
lr	wrap	none	0.754	0.837	0.710	0.719	0.759	0.755	0.710	0.529
sgd	none	none	0.750	0.730	0.730	0.733	0.690	0.788	0.730	0.647
sgd	embed_linear	linear	0.744	0.812	0.724	0.726	0.684	0.783	0.724	0.639
sgd	assoc	none	0.741	0.791	0.716	0.720	0.687	0.775	0.716	0.614
sgd	embed_lgbm	lgbm	0.725	0.814	0.702	0.704	0.649	0.768	0.702	0.605
knn	pred	none	0.716	0.782	0.644	0.640	0.795	0.702	0.644	0.344
knn	none	none	0.697	0.755	0.615	0.599	0.778	0.684	0.615	0.276
knn	embed_linear	linear	0.697	0.755	0.615	0.599	0.778	0.684	0.615	0.276
knn	assoc	none	0.697	0.755	0.615	0.599	0.778	0.684	0.615	0.276
knn	embed_lgbm	lgbm	0.693	0.750	0.656	0.658	0.623	0.728	0.656	0.504
knn	wrap	none	0.690	0.718	0.658	0.660	0.617	0.731	0.658	0.522
dummy	embed_lgbm	lgbm	0.620	0.500	0.500	0.383	nan	0.620	0.500	0.000
dummy	embed_linear	linear	0.620	0.500	0.500	0.383	nan	0.620	0.500	0.000
dummy	wrap	none	0.620	0.500	0.500	0.383	nan	0.620	0.500	0.000
dummy	none	none	0.620	0.500	0.500	0.383	nan	0.620	0.500	0.000
dummy	pred	none	0.620	0.500	0.500	0.383	nan	0.620	0.500	0.000
dummy	assoc	none	0.620	0.500	0.500	0.383	nan	0.620	0.500	0.000

nan = Not a Number.

## Appendix G

Filter-based Association Feature Selection Results for Predicting Management with US image derived features.

**Table A18:** Continuous Feature scores (Mutual Information: Higher = More important).

	Mut_Info
CRP__Management.0	1.396 × 10^−1^
CRP__Management.1	1.358 × 10^−1^
Alvarado_Score__Management.1	1.052 × 10^−1^
Appendix_Diameter__Management.0	9.615 × 10^−2^
Appendix_Diameter__Management.1	8.457 × 10^−2^
WBC_Count__Management.1	7.696 × 10^−2^
Neutrophil_Percentage__Management.0	7.531 × 10^−2^
Paedriatic_Appendicitis_Score__Management.0	7.084 × 10^−2^
Neutrophil_Percentage__Management.1	6.487 × 10^−2^
Alvarado_Score__Management.0	6.183 × 10^−2^
WBC_Count__Management.0	6.059 × 10^−2^
Height__Management.1	5.915 × 10^−2^
RDW__Management.0	5.379 × 10^−2^
Segmented_Neutrophils__Management.0	5.172 × 10^−2^
Paedriatic_Appendicitis_Score__Management.1	4.978 × 10^−2^
RDW__Management.1	4.709 × 10^−2^
Ketones_in_Urine__Management.0	4.477 × 10^−2^
Weight__Management.0	4.001 × 10^−2^
Weight__Management.1	3.639 × 10^−2^
Hemoglobin__Management.0	3.513 × 10^−2^
Height__Management.0	3.048 × 10^−2^
Body_Temperature__Management.0	2.882 × 10^−2^
Ketones_in_Urine__Management.1	2.756 × 10^−2^
RBC_Count__Management.1	1.856 × 10^−2^
Body_Temperature__Management.1	1.781 × 10^−2^
Hemoglobin__Management.1	1.754 × 10^−2^
WBC_in_Urine__Management.1	1.720 × 10^−2^
RBC_Count__Management.0	1.283 × 10^−2^
Age__Management.0	9.069 × 10^−3^
Age__Management.1	8.695 × 10^−3^
RBC_in_Urine__Management.1	8.061 × 10^−3^
Segmented_Neutrophils__Management.1	0.000 × 10^0^
Thrombocyte_Count__Management.0	0.000 × 10^0^
Thrombocyte_Count__Management.1	0.000 × 10^0^
BMI__Management.1	0.000 × 10^0^
RBC_in_Urine__Management.0	0.000 × 10^0^
WBC_in_Urine__Management.0	0.000 × 10^0^
BMI__Management.0	0.000 × 10^0^

**Table A19:** Categorical Feature scores (Mutual Information: Higher = More important).

Feature_Targetclass	Mut_Info
Ipsilateral_Rebound_Tenderness__Management.surgical	2.827 × 10^−1^
Ipsilateral_Rebound_Tenderness__Management.conservative	2.827 × 10^−1^
Ipsilateral_Rebound_Tenderness	2.827 × 10^−1^
Diagnosis	2.553 × 10^−1^
Diagnosis__Management.surgical	2.553 × 10^−1^
Diagnosis__Management.conservative	2.553 × 10^−1^
Peritonitis__Management.conservative	1.963 × 10^−1^
Peritonitis	1.963 × 10^−1^
Peritonitis__Management.surgical	1.963 × 10^−1^
Severity	1.800 × 10^−1^
Severity__Management.conservative	1.800 × 10^−1^
Severity__Management.surgical	1.800 × 10^−1^
Surrounding_Tissue_Reaction__Management.conservative	1.077 × 10^−1^
Surrounding_Tissue_Reaction	1.077 × 10^−1^
Surrounding_Tissue_Reaction__Management.surgical	1.077 × 10^−1^
Neutrophilia__Management.surgical	6.087 × 10^−2^
Neutrophilia	6.087 × 10^−2^
Neutrophilia__Management.conservative	6.087 × 10^−2^
Appendix_Wall_Layers__Management.conservative	5.302 × 10^−2^
Appendix_Wall_Layers	5.302 × 10^−2^
Appendix_Wall_Layers__Management.surgical	5.302 × 10^−2^
Ileus__Management.conservative	4.696 × 10^−2^
Ileus__Management.surgical	4.696 × 10^−2^
Ileus	4.696 × 10^−2^
Dysuria__Management.conservative	3.966 × 10^−2^
Dysuria	3.966 × 10^−2^
Dysuria__Management.surgical	3.966 × 10^−2^
Free_Fluids__Management.surgical	3.871 × 10^−2^
Free_Fluids__Management.conservative	3.871 × 10^−2^
Free_Fluids	3.871 × 10^−2^
Perforation__Management.conservative	3.749 × 10^−2^
Perforation__Management.surgical	3.749 × 10^−2^
Perforation	3.749 × 10^−2^
Appendicolith	3.328 × 10^−2^
Appendicolith__Management.surgical	3.328 × 10^−2^
Appendicolith__Management.conservative	3.328 × 10^−2^
Psoas_Sign	3.245 × 10^−2^
Psoas_Sign__Management.surgical	3.245 × 10^−2^
Psoas_Sign__Management.conservative	3.245 × 10^−2^
Target_Sign__Management.surgical	3.087 × 10^−2^
Target_Sign__Management.conservative	3.087 × 10^−2^
Target_Sign	3.087 × 10^−2^
Contralateral_Rebound_Tenderness	2.795 × 10^−2^
Contralateral_Rebound_Tenderness__Management.surgical	2.795 × 10^−2^
Contralateral_Rebound_Tenderness__Management.conservative	2.795 × 10^−2^
Coprostasis__Management.conservative	2.749 × 10^−2^
Coprostasis	2.749 × 10^−2^
Coprostasis__Management.surgical	2.749 × 10^−2^
Perfusion__Management.conservative	2.600 × 10^−2^
Perfusion__Management.surgical	2.600 × 10^−2^
Perfusion	2.600 × 10^−2^
Nausea	2.583 × 10^−2^
Nausea__Management.surgical	2.583 × 10^−2^
Nausea__Management.conservative	2.583 × 10^−2^
Loss_of_Appetite__Management.surgical	2.467 × 10^−2^
Loss_of_Appetite__Management.conservative	2.467 × 10^−2^
Loss_of_Appetite	2.467 × 10^−2^
Enteritis__Management.surgical	2.135 × 10^−2^
Enteritis	2.135 × 10^−2^
Enteritis__Management.conservative	2.135 × 10^−2^
Stool__Management.conservative	2.133 × 10^−2^
Stool__Management.surgical	2.133 × 10^−2^
Stool	2.133 × 10^−2^
Conglomerate_of_Bowel_Loops__Management.conservative	2.066 × 10^−2^
Conglomerate_of_Bowel_Loops__Management.surgical	2.066 × 10^−2^
Conglomerate_of_Bowel_Loops	2.066 × 10^−2^
Bowel_Wall_Thickening__Management.surgical	1.697 × 10^−2^
Bowel_Wall_Thickening__Management.conservative	1.697 × 10^−2^
Bowel_Wall_Thickening	1.697 × 10^−2^
Appendicular_Abscess	1.666 × 10^−2^
Appendicular_Abscess__Management.conservative	1.666 × 10^−2^
Appendicular_Abscess__Management.surgical	1.666 × 10^−2^
Coughing_Pain__Management.conservative	1.565 × 10^−2^
Coughing_Pain__Management.surgical	1.565 × 10^−2^
Coughing_Pain	1.565 × 10^−2^
Meteorism	1.319 × 10^−2^
Meteorism__Management.surgical	1.319 × 10^−2^
Meteorism__Management.conservative	1.319 × 10^−2^
Appendix_on_US	1.000 × 10^−2^
Appendix_on_US__Management.conservative	1.000 × 10^−2^
Appendix_on_US__Management.surgical	1.000 × 10^−2^
US_Performed	6.948 × 10^−3^
US_Performed__Management.surgical	6.948 × 10^−3^
US_Performed__Management.conservative	6.948 × 10^−3^
Lower_Right_Abd_Pain__Management.conservative	6.616 × 10^−3^
Lower_Right_Abd_Pain__Management.surgical	6.616 × 10^−3^
Lower_Right_Abd_Pain	6.616 × 10^−3^
Migratory_Pain__Management.surgical	6.200 × 10^−3^
Migratory_Pain	6.200 × 10^−3^
Migratory_Pain__Management.conservative	6.200 × 10^−3^
Pathological_Lymph_Nodes__Management.conservative	5.161 × 10^−3^
Pathological_Lymph_Nodes__Management.surgical	5.161 × 10^−3^
Pathological_Lymph_Nodes	5.161 × 10^−3^
Sex__Management.conservative	4.313 × 10^−4^
Sex__Management.surgical	4.313 × 10^−4^
Sex	4.313 × 10^−4^

## Appendix H

Results from predicting management without US image features.

**Table A20:** Management without US image features holdout set performance.

Model	Selection	Embed_Selector	Acc	Auroc	Bal-Acc	F1	Npv	Ppv	Sens	Spec
rf	none	none	0.939	0.980	0.925	0.934	0.972	0.923	0.925	0.866
rf	assoc	none	0.936	0.980	0.922	0.931	0.963	0.922	0.922	0.866
lgbm	embed_linear	linear	0.936	0.977	0.921	0.930	0.971	0.918	0.921	0.857
lgbm	embed_lgbm	lgbm	0.933	0.975	0.918	0.927	0.962	0.918	0.918	0.857
rf	embed_linear	linear	0.933	0.979	0.918	0.927	0.962	0.918	0.918	0.857
lgbm	none	none	0.930	0.978	0.916	0.924	0.953	0.917	0.916	0.857
rf	embed_lgbm	lgbm	0.930	0.979	0.916	0.924	0.953	0.917	0.916	0.857
lgbm	assoc	none	0.930	0.977	0.914	0.924	0.962	0.913	0.914	0.849
rf	pred	none	0.927	0.981	0.911	0.920	0.953	0.913	0.911	0.849
rf	wrap	none	0.920	0.960	0.901	0.913	0.961	0.900	0.901	0.824
lgbm	pred	none	0.917	0.978	0.896	0.909	0.970	0.893	0.896	0.807
lgbm	wrap	none	0.917	0.953	0.894	0.908	0.979	0.889	0.894	0.798
lr	pred	none	0.856	0.932	0.819	0.836	0.940	0.825	0.819	0.664
lr	none	none	0.837	0.925	0.802	0.816	0.886	0.818	0.802	0.655
lr	embed_linear	linear	0.837	0.925	0.802	0.816	0.886	0.818	0.802	0.655
lr	assoc	none	0.837	0.925	0.802	0.816	0.886	0.818	0.802	0.655
sgd	none	none	0.834	0.822	0.822	0.823	0.786	0.862	0.822	0.773
lr	embed_lgbm	lgbm	0.824	0.916	0.788	0.802	0.864	0.809	0.788	0.639
sgd	assoc	none	0.824	0.813	0.813	0.813	0.771	0.856	0.813	0.765
lr	wrap	none	0.815	0.903	0.771	0.786	0.886	0.791	0.771	0.588
sgd	pred	none	0.802	0.788	0.788	0.789	0.744	0.837	0.788	0.731
sgd	embed_linear	linear	0.767	0.862	0.744	0.748	0.713	0.795	0.744	0.647
sgd	embed_lgbm	lgbm	0.754	0.750	0.738	0.739	0.678	0.800	0.738	0.672
knn	assoc	none	0.719	0.732	0.666	0.671	0.707	0.723	0.666	0.445
knn	embed_linear	linear	0.719	0.732	0.666	0.671	0.707	0.723	0.666	0.445
knn	wrap	none	0.709	0.723	0.642	0.642	0.741	0.702	0.642	0.361
knn	pred	none	0.706	0.748	0.633	0.629	0.765	0.695	0.633	0.328
sgd	wrap	none	0.703	0.802	0.677	0.680	0.618	0.749	0.677	0.571
knn	embed_lgbm	lgbm	0.703	0.739	0.653	0.657	0.662	0.717	0.653	0.445
knn	none	none	0.626	0.588	0.588	0.589	0.510	0.681	0.588	0.429
dummy	embed_lgbm	lgbm	0.620	0.500	0.500	0.383	nan	0.620	0.500	0.000
dummy	wrap	none	0.620	0.500	0.500	0.383	nan	0.620	0.500	0.000
dummy	pred	none	0.620	0.500	0.500	0.383	nan	0.620	0.500	0.000
dummy	none	none	0.620	0.500	0.500	0.383	nan	0.620	0.500	0.000
dummy	embed_linear	linear	0.620	0.500	0.500	0.383	nan	0.620	0.500	0.000
dummy	assoc	none	0.620	0.500	0.500	0.383	nan	0.620	0.500	0.000

nan = Not a Number.

**Table A21:** Management without US image features 5-fold performance on the holdout set.

Model	Selection	Embed_Selector	Acc	Auroc	Bal-Acc	F1	Npv	Ppv	Sens	Spec
lgbm	none	none	0.930	0.971	0.916	0.923	0.952	0.919	0.916	0.858
rf	wrap	none	0.927	0.942	0.914	0.920	0.945	0.920	0.914	0.858
rf	embed_lgbm	lgbm	0.927	0.968	0.912	0.920	0.952	0.914	0.912	0.850
lgbm	assoc	none	0.920	0.973	0.905	0.913	0.944	0.910	0.905	0.841
rf	assoc	none	0.920	0.969	0.905	0.913	0.943	0.909	0.905	0.841
rf	none	none	0.920	0.970	0.907	0.913	0.934	0.913	0.907	0.849
lgbm	embed_linear	linear	0.917	0.970	0.903	0.909	0.935	0.910	0.903	0.841
lgbm	pred	none	0.914	0.974	0.905	0.907	0.903	0.922	0.905	0.867
rf	embed_linear	linear	0.914	0.964	0.898	0.906	0.933	0.904	0.898	0.833
rf	pred	none	0.907	0.968	0.892	0.899	0.925	0.900	0.892	0.825
lgbm	embed_lgbm	lgbm	0.898	0.960	0.885	0.889	0.895	0.905	0.885	0.833
lgbm	wrap	none	0.895	0.948	0.875	0.884	0.927	0.884	0.875	0.791
lr	pred	none	0.776	0.863	0.743	0.751	0.767	0.785	0.743	0.605
lr	assoc	none	0.760	0.864	0.725	0.733	0.735	0.772	0.725	0.580
lr	none	none	0.760	0.864	0.725	0.733	0.735	0.772	0.725	0.580
lr	embed_linear	linear	0.760	0.864	0.725	0.733	0.735	0.772	0.725	0.580
lr	wrap	none	0.751	0.841	0.713	0.721	0.728	0.761	0.713	0.554
sgd	pred	none	0.751	0.735	0.735	0.735	0.675	0.800	0.735	0.672
lr	embed_lgbm	lgbm	0.748	0.854	0.713	0.720	0.708	0.766	0.713	0.571
sgd	none	none	0.738	0.722	0.722	0.722	0.661	0.789	0.722	0.656
sgd	embed_linear	linear	0.735	0.815	0.718	0.718	0.657	0.784	0.718	0.647
sgd	wrap	none	0.732	0.787	0.708	0.711	0.665	0.773	0.708	0.613
sgd	assoc	none	0.731	0.715	0.715	0.715	0.659	0.782	0.715	0.647
sgd	embed_lgbm	lgbm	0.709	0.712	0.691	0.691	0.626	0.763	0.691	0.614
knn	wrap	none	0.700	0.721	0.639	0.638	0.693	0.705	0.639	0.386
knn	pred	none	0.697	0.759	0.612	0.592	0.812	0.681	0.612	0.260
knn	embed_lgbm	lgbm	0.684	0.723	0.648	0.650	0.610	0.722	0.648	0.496
knn	embed_linear	linear	0.658	0.708	0.622	0.622	0.571	0.705	0.622	0.471
knn	assoc	none	0.658	0.708	0.622	0.622	0.571	0.705	0.622	0.471
knn	none	none	0.636	0.620	0.620	0.617	0.522	0.715	0.620	0.555
dummy	embed_lgbm	lgbm	0.620	0.500	0.500	0.383	nan	0.620	0.500	0.000
dummy	wrap	none	0.620	0.500	0.500	0.383	nan	0.620	0.500	0.000
dummy	pred	none	0.620	0.500	0.500	0.383	nan	0.620	0.500	0.000
dummy	none	none	0.620	0.500	0.500	0.383	nan	0.620	0.500	0.000
dummy	embed_linear	linear	0.620	0.500	0.500	0.383	nan	0.620	0.500	0.000
dummy	assoc	none	0.620	0.500	0.500	0.383	nan	0.620	0.500	0.000

nan = Not a Number.

## Appendix I

Redundancy-Aware Step-Up Feature Selection Results for predicting Management without US image features.

**Table A22:** Selection scores (Accuracy: Higher = More important) for redundancy-aware feature selection predicting management without US features.

Feature	Score
Ipsilateral_Rebound_Tenderness_nan	8.312 × 10^−1^
Severity_uncomplicated	8.932 × 10^−1^
RDW	8.953 × 10^−1^
Peritonitis_no	9.359 × 10^−1^
WBC_Count	9.359 × 10^−1^
Peritonitis_local	9.295 × 10^−1^
Body_Temperature	9.274 × 10^−1^
Weight	8.932 × 10^−1^
CRP	8.720 × 10^−1^
Segmented_Neutrophils	8.397 × 10^−1^
Height	7.884 × 10^−1^
Thrombocyte_Count	7.478 × 10^−1^

## Appendix J

Results from predicting Severity with US image features.

**Table A23:** Severity with US image features holdout set performance.

Model	Selection	Embed_Selector	Acc	Auroc	Bal-Acc	F1	Npv	Ppv	Sens	Spec
lgbm	pred	none	0.891	0.908	0.723	0.756	0.911	0.719	0.723	0.966
lr	wrap	none	0.891	0.834	0.706	0.745	0.905	0.750	0.706	0.974
lr	pred	none	0.891	0.879	0.706	0.745	0.905	0.750	0.706	0.974
lgbm	embed_lgbm	lgbm	0.891	0.939	0.782	0.787	0.933	0.652	0.782	0.940
lr	assoc	none	0.888	0.893	0.704	0.741	0.905	0.724	0.704	0.970
sgd	assoc	none	0.888	0.854	0.712	0.746	0.908	0.710	0.712	0.966
rf	embed_lgbm	lgbm	0.888	0.929	0.780	0.783	0.932	0.638	0.780	0.936
lr	none	none	0.888	0.895	0.704	0.741	0.905	0.724	0.704	0.970
lgbm	assoc	none	0.888	0.932	0.755	0.771	0.923	0.659	0.755	0.947
rf	wrap	none	0.885	0.879	0.727	0.753	0.913	0.667	0.727	0.955
rf	pred	none	0.885	0.906	0.753	0.766	0.923	0.643	0.753	0.943
lr	embed_linear	linear	0.885	0.892	0.702	0.736	0.905	0.700	0.702	0.966
sgd	none	none	0.882	0.857	0.700	0.732	0.904	0.677	0.700	0.962
sgd	embed_linear	linear	0.882	0.864	0.700	0.732	0.904	0.677	0.700	0.962
rf	assoc	none	0.882	0.933	0.811	0.788	0.945	0.596	0.811	0.913
lr	embed_lgbm	lgbm	0.882	0.883	0.691	0.726	0.901	0.690	0.691	0.966
sgd	wrap	none	0.882	0.825	0.700	0.732	0.904	0.677	0.700	0.962
lgbm	none	none	0.882	0.933	0.751	0.762	0.922	0.628	0.751	0.940
lgbm	embed_linear	linear	0.882	0.930	0.743	0.758	0.919	0.634	0.743	0.943
knn	none	none	0.882	0.833	0.674	0.713	0.896	0.720	0.674	0.974
knn	embed_linear	linear	0.882	0.788	0.691	0.726	0.901	0.690	0.691	0.966
knn	embed_lgbm	lgbm	0.882	0.826	0.666	0.706	0.893	0.739	0.666	0.977
knn	assoc	none	0.882	0.788	0.691	0.726	0.901	0.690	0.691	0.966
rf	embed_linear	linear	0.879	0.933	0.809	0.784	0.945	0.586	0.809	0.909
sgd	pred	none	0.879	0.846	0.698	0.728	0.904	0.656	0.698	0.958
rf	none	none	0.872	0.920	0.780	0.766	0.934	0.574	0.780	0.913
sgd	embed_lgbm	lgbm	0.872	0.846	0.720	0.736	0.912	0.600	0.720	0.940
lgbm	wrap	none	0.869	0.869	0.709	0.726	0.909	0.590	0.709	0.940
knn	wrap	none	0.869	0.753	0.650	0.682	0.889	0.640	0.650	0.966
knn	pred	none	0.856	0.743	0.625	0.651	0.882	0.560	0.625	0.958
dummy	embed_lgbm	lgbm	0.847	0.500	0.500	0.458	0.847	nan	0.500	1.000
dummy	wrap	none	0.847	0.500	0.500	0.458	0.847	nan	0.500	1.000
dummy	pred	none	0.847	0.500	0.500	0.458	0.847	nan	0.500	1.000
dummy	none	none	0.847	0.500	0.500	0.458	0.847	nan	0.500	1.000
dummy	embed_linear	linear	0.847	0.500	0.500	0.458	0.847	nan	0.500	1.000
dummy	assoc	none	0.847	0.500	0.500	0.458	0.847	nan	0.500	1.000

nan = Not a Number.

**Table A24:** Severity with US image features 5-fold performance on the holdout set.

Model	Selection	Embed_Selector	Acc	Auroc	Bal-Acc	F1	Npv	Ppv	Sens	Spec
lr	wrap	none	0.895	0.820	0.699	0.744	0.903	0.813	0.699	0.981
lr	assoc	none	0.891	0.873	0.707	0.746	0.905	0.753	0.707	0.974
lr	none	none	0.891	0.875	0.707	0.746	0.905	0.753	0.707	0.974
lr	embed_lgbm	lgbm	0.891	0.873	0.707	0.746	0.905	0.753	0.707	0.974
lr	embed_linear	linear	0.891	0.870	0.707	0.746	0.905	0.753	0.707	0.974
rf	assoc	none	0.888	0.926	0.790	0.785	0.936	0.628	0.790	0.932
sgd	assoc	none	0.888	0.845	0.696	0.735	0.902	0.747	0.696	0.974
knn	pred	none	0.888	0.767	0.695	0.734	0.902	0.750	0.695	0.974
rf	embed_linear	linear	0.885	0.924	0.789	0.781	0.936	0.617	0.789	0.928
lr	pred	none	0.885	0.856	0.687	0.724	0.899	0.753	0.687	0.974
sgd	embed_lgbm	lgbm	0.885	0.842	0.711	0.742	0.908	0.695	0.711	0.962
sgd	embed_linear	linear	0.885	0.855	0.695	0.730	0.902	0.716	0.695	0.970
rf	none	none	0.885	0.924	0.770	0.775	0.929	0.632	0.770	0.936
sgd	none	none	0.885	0.845	0.695	0.730	0.902	0.716	0.695	0.970
lgbm	wrap	none	0.885	0.837	0.694	0.730	0.902	0.713	0.694	0.970
knn	none	none	0.882	0.781	0.649	0.688	0.888	0.800	0.649	0.985
rf	pred	none	0.879	0.874	0.707	0.735	0.907	0.660	0.707	0.955
rf	wrap	none	0.879	0.856	0.707	0.734	0.907	0.656	0.707	0.955
sgd	pred	none	0.879	0.840	0.692	0.719	0.902	0.699	0.692	0.962
lgbm	pred	none	0.876	0.867	0.693	0.714	0.902	0.642	0.693	0.958
lgbm	embed_linear	linear	0.876	0.916	0.675	0.701	0.896	0.664	0.675	0.966
sgd	wrap	none	0.876	0.824	0.689	0.718	0.901	0.652	0.689	0.958
lgbm	embed_lgbm	lgbm	0.876	0.924	0.714	0.733	0.910	0.629	0.714	0.947
rf	embed_lgbm	lgbm	0.872	0.928	0.645	0.676	0.887	0.680	0.645	0.974
knn	embed_linear	linear	0.872	0.718	0.634	0.667	0.884	0.733	0.634	0.977
knn	assoc	none	0.872	0.718	0.634	0.667	0.884	0.733	0.634	0.977
lgbm	none	none	0.866	0.897	0.651	0.677	0.889	0.600	0.651	0.962
lgbm	assoc	none	0.866	0.915	0.615	0.644	0.878	0.687	0.615	0.977
knn	wrap	none	0.866	0.716	0.648	0.675	0.889	0.668	0.648	0.962
dummy	embed_lgbm	lgbm	0.847	0.500	0.500	0.458	0.847	nan	0.500	1.000
knn	embed_lgbm	lgbm	0.847	0.815	0.500	0.458	0.847	nan	0.500	1.000
dummy	wrap	none	0.847	0.500	0.500	0.458	0.847	nan	0.500	1.000
dummy	pred	none	0.847	0.500	0.500	0.458	0.847	nan	0.500	1.000
dummy	none	none	0.847	0.500	0.500	0.458	0.847	nan	0.500	1.000
dummy	embed_linear	linear	0.847	0.500	0.500	0.458	0.847	nan	0.500	1.000
dummy	assoc	none	0.847	0.500	0.500	0.458	0.847	nan	0.500	1.000

nan = Not a Number.

## Appendix K

Redundancy-Aware Step-Up Feature Selection Results for predicting Severity with US image features.

**Table A25:** Selection scores (Accuracy: Higher = More important) for redundancy-aware feature selection predicting severity with US features.

Feature	Score
CRP	8.697 × 10^−1^
Peritonitis_no	8.846 × 10^−1^
Neutrophil_Percentage	8.889 × 10^−1^
Thrombocyte_Count	8.954 × 10^−1^
Weight_NAN	8.996 × 10^−1^
Dysuria_nan	8.996 × 10^−1^
Meteorism_nan	8.997 × 10^−1^
Lower_Right_Abd_Pain_nan	8.975 × 10^−1^
Free_Fluids_nan	8.975 × 10^−1^
Nausea_nan	8.954 × 10^−1^
Lower_Right_Abd_Pain_yes	8.932 × 10^−1^
Peritonitis_generalized	8.846 × 10^−1^
Segmented_Neutrophils	8.740 × 10^−1^
Height	8.654 × 10^−1^

## Appendix L

Results from predicting Severity without US image features.

**Table A26:** Severity without US image features holdout set performance.

Model	Selection	Embed_Selector	Acc	Auroc	Bal-Acc	F1	Npv	Ppv	Sens	Spec
lgbm	assoc	none	0.901	0.931	0.788	0.801	0.933	0.698	0.788	0.951
rf	assoc	none	0.891	0.933	0.782	0.787	0.933	0.652	0.782	0.940
lr	wrap	none	0.888	0.811	0.695	0.735	0.902	0.741	0.695	0.974
lr	pred	none	0.888	0.881	0.695	0.735	0.902	0.741	0.695	0.974
lr	embed_lgbm	lgbm	0.888	0.878	0.695	0.735	0.902	0.741	0.695	0.974
lgbm	none	none	0.888	0.931	0.755	0.771	0.923	0.659	0.755	0.947
lr	assoc	none	0.885	0.889	0.693	0.730	0.902	0.714	0.693	0.970
sgd	pred	none	0.885	0.827	0.676	0.718	0.896	0.750	0.676	0.977
sgd	embed_lgbm	lgbm	0.885	0.854	0.702	0.736	0.905	0.700	0.702	0.966
rf	wrap	none	0.885	0.873	0.702	0.736	0.905	0.700	0.702	0.966
lr	none	none	0.885	0.889	0.693	0.730	0.902	0.714	0.693	0.970
sgd	wrap	none	0.885	0.788	0.685	0.724	0.899	0.731	0.685	0.974
knn	embed_linear	linear	0.885	0.807	0.702	0.736	0.905	0.700	0.702	0.966
sgd	none	none	0.882	0.855	0.700	0.732	0.904	0.677	0.700	0.962
knn	assoc	none	0.882	0.829	0.666	0.706	0.893	0.739	0.666	0.977
lr	embed_linear	linear	0.882	0.889	0.691	0.726	0.901	0.690	0.691	0.966
knn	none	none	0.882	0.795	0.700	0.732	0.904	0.677	0.700	0.962
knn	wrap	none	0.882	0.817	0.666	0.706	0.893	0.739	0.666	0.977
lgbm	embed_linear	linear	0.882	0.929	0.768	0.770	0.929	0.617	0.768	0.932
sgd	assoc	none	0.882	0.855	0.700	0.732	0.904	0.677	0.700	0.962
lgbm	pred	none	0.879	0.928	0.749	0.758	0.922	0.614	0.749	0.936
sgd	embed_linear	linear	0.879	0.817	0.681	0.715	0.898	0.679	0.681	0.966
knn	embed_lgbm	lgbm	0.875	0.772	0.696	0.723	0.904	0.636	0.696	0.955
rf	embed_linear	linear	0.875	0.933	0.781	0.770	0.935	0.585	0.781	0.917
rf	pred	none	0.875	0.930	0.798	0.777	0.941	0.579	0.798	0.909
lgbm	wrap	none	0.872	0.850	0.660	0.693	0.892	0.654	0.660	0.966
rf	embed_lgbm	lgbm	0.872	0.926	0.805	0.776	0.945	0.567	0.805	0.902
rf	none	none	0.872	0.926	0.797	0.773	0.941	0.569	0.797	0.906
lgbm	embed_lgbm	lgbm	0.869	0.930	0.735	0.741	0.918	0.578	0.735	0.928
dummy	embed_linear	linear	0.847	0.500	0.500	0.458	0.847	nan	0.500	1.000
dummy	none	none	0.847	0.500	0.500	0.458	0.847	nan	0.500	1.000
dummy	wrap	none	0.847	0.500	0.500	0.458	0.847	nan	0.500	1.000
dummy	pred	none	0.847	0.500	0.500	0.458	0.847	nan	0.500	1.000
dummy	embed_lgbm	lgbm	0.847	0.500	0.500	0.458	0.847	nan	0.500	1.000
dummy	assoc	none	0.847	0.500	0.500	0.458	0.847	nan	0.500	1.000
knn	pred	none	0.843	0.717	0.618	0.637	0.880	0.483	0.618	0.943

nan = Not a Number.

**Table A27:** Severity without US image features 5-fold performance on the holdout set.

Model	Selection	Embed_Selector	Acc	Auroc	Bal-Acc	F1	Npv	Ppv	Sens	Spec
lgbm	assoc	none	0.892	0.896	0.741	0.768	0.917	0.717	0.741	0.958
sgd	none	none	0.891	0.850	0.699	0.739	0.903	0.783	0.699	0.977
lr	assoc	none	0.891	0.869	0.707	0.745	0.906	0.770	0.707	0.974
lr	embed_lgbm	lgbm	0.891	0.864	0.707	0.745	0.906	0.770	0.707	0.974
lr	none	none	0.891	0.869	0.707	0.745	0.906	0.770	0.707	0.974
lr	embed_linear	linear	0.891	0.869	0.707	0.745	0.906	0.770	0.707	0.974
lgbm	embed_lgbm	lgbm	0.888	0.907	0.721	0.753	0.911	0.709	0.721	0.962
sgd	embed_lgbm	lgbm	0.888	0.846	0.696	0.735	0.902	0.747	0.696	0.974
knn	none	none	0.888	0.746	0.696	0.735	0.902	0.747	0.696	0.974
lr	wrap	none	0.888	0.805	0.686	0.727	0.899	0.767	0.686	0.977
rf	assoc	none	0.885	0.923	0.712	0.742	0.908	0.687	0.712	0.962
sgd	assoc	none	0.885	0.853	0.695	0.730	0.902	0.716	0.695	0.970
rf	wrap	none	0.885	0.821	0.694	0.731	0.902	0.728	0.694	0.970
lr	pred	none	0.885	0.847	0.678	0.717	0.896	0.777	0.678	0.977
sgd	wrap	none	0.885	0.770	0.685	0.724	0.899	0.737	0.685	0.974
sgd	pred	none	0.882	0.780	0.684	0.720	0.899	0.741	0.684	0.970
knn	embed_linear	linear	0.882	0.731	0.691	0.725	0.902	0.702	0.691	0.966
rf	pred	none	0.876	0.921	0.726	0.731	0.914	0.593	0.726	0.943
rf	embed_lgbm	lgbm	0.876	0.918	0.816	0.781	0.949	0.570	0.816	0.902
rf	embed_linear	linear	0.875	0.925	0.689	0.717	0.901	0.645	0.689	0.958
knn	embed_lgbm	lgbm	0.872	0.719	0.634	0.667	0.884	0.733	0.634	0.977
lgbm	embed_linear	linear	0.869	0.928	0.593	0.605	0.872	0.700	0.593	0.992
lgbm	wrap	none	0.869	0.807	0.684	0.709	0.900	0.620	0.684	0.951
knn	pred	none	0.869	0.738	0.658	0.687	0.892	0.634	0.658	0.962
sgd	embed_linear	linear	0.866	0.845	0.711	0.716	0.911	0.601	0.711	0.936
lgbm	none	none	0.856	0.876	0.609	0.629	0.877	0.567	0.609	0.966
lgbm	pred	none	0.847	0.887	0.500	0.458	0.847	nan	0.500	1.000
rf	none	none	0.847	0.906	0.500	0.458	0.847	nan	0.500	1.000
knn	assoc	none	0.847	0.804	0.500	0.458	0.847	nan	0.500	1.000
dummy	wrap	none	0.847	0.500	0.500	0.458	0.847	nan	0.500	1.000
dummy	pred	none	0.847	0.500	0.500	0.458	0.847	nan	0.500	1.000
dummy	none	none	0.847	0.500	0.500	0.458	0.847	nan	0.500	1.000
knn	wrap	none	0.847	0.816	0.500	0.458	0.847	nan	0.500	1.000
dummy	embed_linear	linear	0.847	0.500	0.500	0.458	0.847	nan	0.500	1.000
dummy	embed_lgbm	lgbm	0.847	0.500	0.500	0.458	0.847	nan	0.500	1.000
dummy	assoc	none	0.847	0.500	0.500	0.458	0.847	nan	0.500	1.000

nan = Not a Number.

## Appendix M

Redundancy-Aware Step-Up Feature Selection Results for predicting Severity without US image features.

**Table A28:** Selection scores (Accuracy: Higher = More important) for redundancy-aware feature selection predicting severity without ultrasound features.

Feature	Score
CRP	8.697 × 10^−1^
Peritonitis_no	8.868 × 10^−1^
Coughing_Pain_nan	8.868 × 10^−1^
Body_Temperature	8.847 × 10^−1^
Thrombocyte_Count	8.718 × 10^−1^
